# γδ T cells are effectors of immunotherapy in cancers with HLA class I defects

**DOI:** 10.1038/s41586-022-05593-1

**Published:** 2023-01-11

**Authors:** Natasja L. de Vries, Joris van de Haar, Vivien Veninga, Myriam Chalabi, Marieke E. Ijsselsteijn, Manon van der Ploeg, Jitske van den Bulk, Dina Ruano, Jose G. van den Berg, John B. Haanen, Laurien J. Zeverijn, Birgit S. Geurts, Gijs F. de Wit, Thomas W. Battaglia, Hans Gelderblom, Henk M. W. Verheul, Ton N. Schumacher, Lodewyk F. A. Wessels, Frits Koning, Noel F. C. C. de Miranda, Emile E. Voest

**Affiliations:** 1 Department of Pathology, Leiden University Medical Center, Leiden, The Netherlands; 2 Department of Immunology, Leiden University Medical Center, Leiden, The Netherlands; 3 Department of Molecular Oncology and Immunology, Netherlands Cancer Institute, Amsterdam, The Netherlands; 4 Oncode Institute, Utrecht, The Netherlands; 5 Division of Molecular Carcinogenesis, Netherlands Cancer Institute, Amsterdam, The Netherlands; 6 Gastrointestinal Oncology, Netherlands Cancer Institute, Amsterdam, The Netherlands; 7 Medical Oncology, Netherlands Cancer Institute, Amsterdam, The Netherlands; 8 Department of Pathology, Netherlands Cancer Institute, Amsterdam, The Netherlands; 9 Department of Medical Oncology, Leiden University Medical Center, Leiden, The Netherlands; 10 Department of Medical Oncology, Erasmus MC, Rotterdam, The Netherlands; 11 Department of Hematology, Leiden University Medical Center, Leiden, The Netherlands; 12 Faculty of EEMCS, Delft University of Technology, Delft, The Netherlands

**Keywords:** Colon cancer, Tumour immunology, Cancer immunotherapy, Antigen processing and presentation, Gammadelta T cells

## Abstract

DNA mismatch repair-deficient (MMR-d) cancers present an abundance of neoantigens that is thought to explain their exceptional responsiveness to immune checkpoint blockade (ICB)^1,2^. Here, in contrast to other cancer types^3–5^, we observed that 20 out of 21 (95%) MMR-d cancers with genomic inactivation of β2-microglobulin (encoded by *B2M*) retained responsiveness to ICB, suggesting the involvement of immune effector cells other than CD8^+^ T cells in this context. We next identified a strong association between *B2M* inactivation and increased infiltration by γδ T cells in MMR-d cancers. These γδ T cells mainly comprised the Vδ1 and Vδ3 subsets, and expressed high levels of PD-1, other activation markers, including cytotoxic molecules, and a broad repertoire of killer-cell immunoglobulin-like receptors. In vitro, PD-1^+^ γδ T cells that were isolated from MMR-d colon cancers exhibited enhanced reactivity to human leukocyte antigen (HLA)-class-I-negative MMR-d colon cancer cell lines and *B2M*-knockout patient-derived tumour organoids compared with antigen-presentation-proficient cells. By comparing paired tumour samples from patients with MMR-d colon cancer that were obtained before and after dual PD-1 and CTLA-4 blockade, we found that immune checkpoint blockade substantially increased the frequency of γδ T cells in B2M-deficient cancers. Taken together, these data indicate that γδ T cells contribute to the response to immune checkpoint blockade in patients with HLA-class-I-negative MMR-d colon cancers, and underline the potential of γδ T cells in cancer immunotherapy.

## Abstract (web summary)

γδ T cells contribute to the response to immune checkpoint blockade treatment in patients with HLA-class-I-negative DNA mismatch repair-deficient colon cancers. .

## Main

ICB targeting the PD-1–PD-L1 and/or CTLA-4 axes provides durable clinical benefits to patients who have cancers with MMR-d and high microsatellite instability^6–9^. The exceptional responses of cancers with MMR-d and high microsatellite instability to ICB is thought to be explained by their substantial burden of putative neoantigens, which originate from the extensive accumulation of mutations in their genomes^1,2^. This is consistent with the current view that PD-1 blockade mainly boosts endogenous antitumour immunity driven by CD8^+^ T cells, which recognize HLA-class-I-bound neoepitopes on cancer cells^10–12^. However, MMR-d colon cancers frequently lose HLA-class-I-mediated antigen presentation due to silencing of HLA class I genes, inactivating mutations in β2-microglobulin (encoded by *B2M*) or other defects in the antigen processing machinery^13–16^, which can render these tumours resistant to CD8^+^ T-cell-mediated immunity^3–5,17^. Notably, early evidence has indicated that B2M-deficient, MMR-d cancers can obtain durable responses to PD-1 blockade^18^, suggesting that immune cell subsets other than CD8^+^ T cells contribute to these responses.

HLA-class-I-unrestricted immune cell subsets, which have the ability to kill tumour cells, include natural killer (NK) cells and γδ T cells. γδ T cells share many characteristics with their αβ T cell counterpart, such as cytotoxic effector functions, but express a distinct TCR that is composed of a γ and a δ chain. Different subsets of γδ T cells are defined by their TCR δ chain use, of which those expressing Vδ1 and Vδ3 are primarily ‘tissue-resident’ at mucosal sites, whereas those expressing Vδ2 are mainly found in blood^19^. Both adaptive and innate mechanisms of activation—for example, through stimulation of their γδ TCR or innate receptors such as NKG2D, DNAM-1, NKp30 or NKp44—have been described for γδ T cells^20^. Killer-cell immunoglobulin-like receptors (KIRs) are expressed by γδ T cells and regulate their activity depending on HLA class I expression in target cells^21^. Furthermore, γδ T cells were found to express high levels of PD-1 in MMR-d colorectal cancers (CRCs)^22^, suggesting that these cells may be targeted by PD-1 blockade.

Here, we applied a combination of transcriptomic and imaging approaches for an in-depth analysis of ICB-naive and ICB-treated MMR-d colon cancers, as well as in vitro functional assays, and found evidence indicating that γδ T cells mediate responses to HLA-class-I-negative MMR-d tumours during treatment with ICB.

## ICB is effective in *B2M*^*MUT*^ MMR-d cancers

**Fig. 1:**
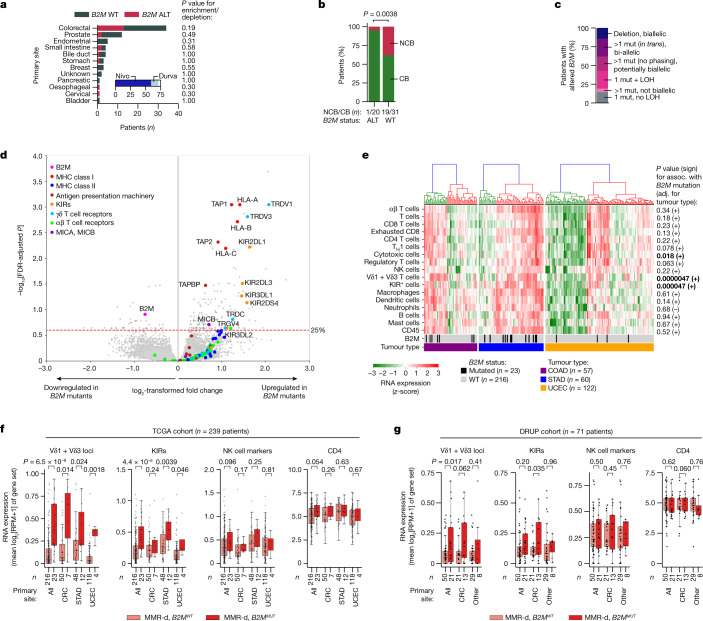
In MMR-d cancers, *B2M* defects are positively associated with ICB responsiveness and infiltration by Vδ1 and Vδ3 T cells and KIR-expressing cells. **a**, Tumour type distribution in the DRUP cohort (*n* = 71 patients). The colours denote patients’ *B2M* status; grey, WT; red, altered (ALT). *P* values for the enrichment/depletion of *B2M*-altered tumours per primary site were calculated using two-sided Fisher’s exact tests. The inset denotes the ICB treatment; dark blue, nivolumab (Nivo); light blue, durvalumab (Durva). **b**, *B2M* status (*x* axis) versus clinical benefit (green, CB; red, no clinical benefit (NCB)) of ICB treatment in the DRUP cohort. The *P* value was calculated using a two-sided Fisher’s exact test. **c**, The allelic status of *B2M* alterations in the DRUP cohort. Mut, mutation. **d**, Differential gene expression between *B2M*^*MUT*^ and *B2M*^*WT*^ MMR-d cancers in the TCGA COAD (colon adenocarcinoma; *n* = 57 patients), STAD (stomach adenocarcinoma; *n* = 60 patients) and UCEC (uterus corpus endometrial carcinoma; *n* = 122 patients) cohorts. The results were adjusted (adj.) for tumour type and multiple-hypothesis testing (Methods). **e**, Immune marker gene set expression in MMR-d cancers of the COAD, STAD and UCEC cohorts of the TCGA. The bottom two bars indicate *B2M* status and cancer type. The association (assoc.) between gene set expression and *B2M* status was tested using ordinary least squares linear regression (adjusted for tumour type; Methods), of which two-sided *P* values and the association sign are shown on the right. Cancers were ranked on the basis of hierarchical clustering (top dendrograms). *P* values less than 0.05 are in bold. **f**, Immune marker gene set expression in *B2M*^*WT*^ (pink) and *B2M*^*MUT*^ (red) MMR-d cancers in the TCGA COAD, STAD and UCEC cohorts separately or combined (all). Boxes, whiskers and dots indicate the quartiles, 1.5× the interquartile range (IQR) and individual data points, respectively. *P* values were calculated using two-sided Wilcoxon rank-sum tests. **g**, Immune marker gene set expression in *B2M*^*WT*^ (pink) and *B2M*^*MUT*^ (red) as described in **f**, but for MMR-d cancers in the DRUP cohort. Results are shown for all cancers combined, only CRC or all non-CRC cancers (other). Two-sided *P* values were calculated using linear regression, adjusting for biopsy site and tumour type (Methods).

**Extended Data Fig. 1:**
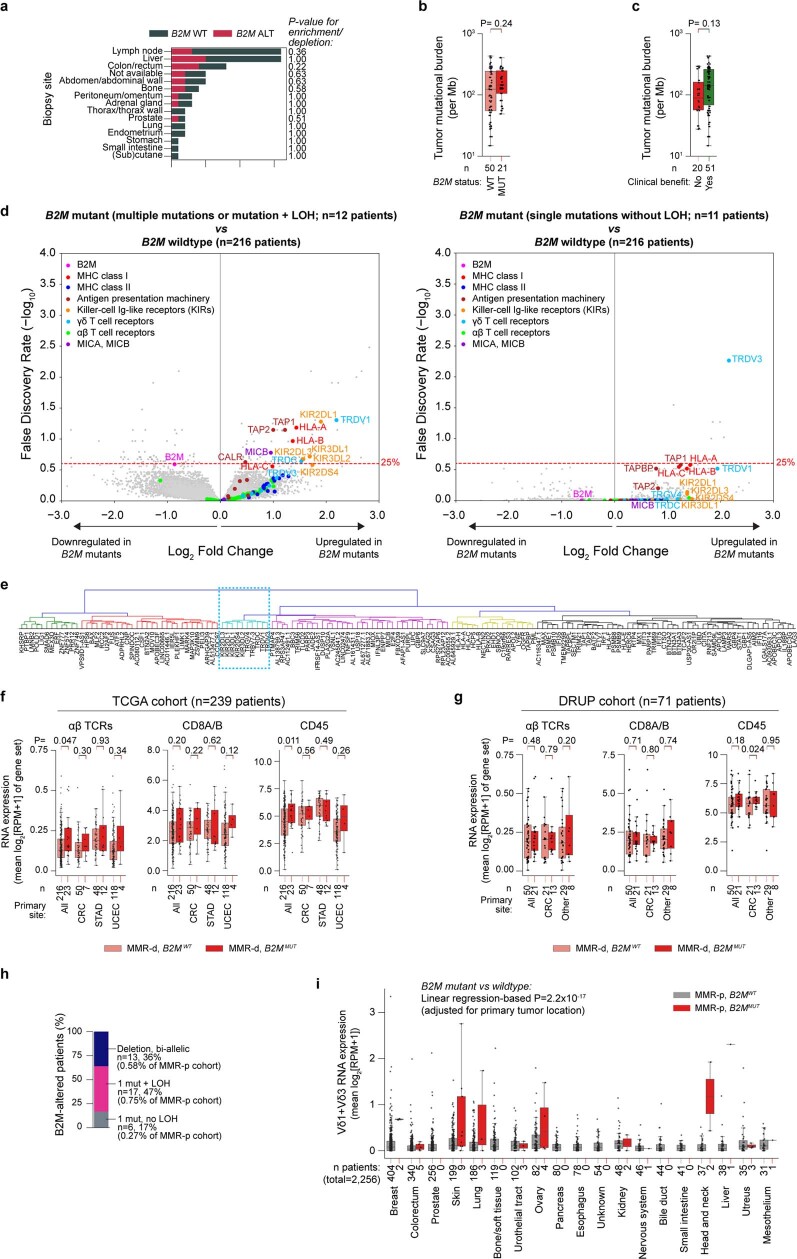
Association of *B2M* status to clinical, genomic, and transcriptomic characteristics of MMR-d and MMR-p tumours. **a**. Biopsy site distribution in the DRUP cohort (n = 71 patients). Colours denote patients’ *B2M* status (WT: wildtype,; ALT: altered). Fisher’s exact test-based two-sided P-values for enrichment/depletion of *B2M* altered tumours per biopsy site are shown. **b**. Tumour mutational burden *vs B2M* status. Wilcoxon rank sum test-based two-sided P-value is shown. Boxes, whiskers, and dots indicate quartiles, 1.5 interquartile ranges, and individual data points, respectively. **c**. As **b**, but for clinical benefit to ICB (x-axis). **d**. Volcano plots indicating differential gene expression between B2M-based subgroups (see title) of MMR-d cancers in TCGA COAD (colon adenocarcinoma; n = 57 patients), STAD (stomach adenocarcinoma; n = 60 patients) and UCEC (uterus corpus endometrial carcinoma; n = 122 patients) cohorts. Results were adjusted for tumour type and multiple hypothesis testing (Methods). **e**. Hierarchical clusters of expression of genes significantly (FDR <25%; see Fig. 1D) upregulated in MMR-d *B2M*^*MUT*^ *vs* MMR-d *B2M*^*WT*^ cancers in the TCGA COAD/STAD/UCEC cohorts. The blue dashed rectangle denotes the Vδ1/3 T cell cluster. **f**. Immune marker gene set expression in *B2M*^*WT*^ (pink), and *B2M*^*MUT*^ (red) MMR-d cancers in the TCGA COAD/STAD/UCEC cohorts separately or combined (All). Boxes, whiskers, and dots indicate quartiles, 1.5 interquartile ranges, and individual data points, respectively. Wilcoxon rank sum test-based two-sided P-values are shown. **g**. As **f**, but for MMR-d cancers in the DRUP cohort, for all cancers combined (All), only colorectal cancer (CRC), or all non-CRC cancers (Other). Two-sided P-values were calculated with linear regression adjusting for biopsy site and tumour type (Methods). **h**. Allelic alteration status of *B2M* in the Hartwig cohort of MMR-p cancers. **i**. RNA expression of Vδ1+Vδ3 loci in MMR-p *B2M*^*WT*^ (grey), and MMR-p *B2M*^*MUT*^ (red) cancers in the Hartwig cohort, stratified per primary tumour location. Boxes, whiskers, and dots indicate quartiles, 1.5 interquartile ranges, and individual data points, respectively. The linear regression-based, two-sided, primary tumour location-adjusted P-value for association of *B2M* status with Vδ1+Vδ3 loci expression is shown.

We evaluated responses to PD-1 blockade therapy in a cohort of 71 patients with MMR-d cancers from various anatomical sites treated in the Drug Rediscovery Protocol (DRUP)^23^ in relation to their *B2M* status (Fig. 1a, Extended Data Fig. 1a–c and Supplementary Table 1). A clinical benefit (CB; defined as at least 4 months of disease control; the primary outcome of the DRUP) was observed in 20 out of 21 (95%) of patients with tumours with mutant or deleted *B2M* (*B2M*^*MUT*^) tumours versus 31 out of 50 (62%) of patients with tumours with wild-type *B2M* (*B2M*^*WT*^) (two-sided Fisher’s exact test, *P* = 0.0038; logistic regression, *P* = 0.022 and *P* = 0.027, adjusted for tumour mutational burden (TMB), and TMB plus tumour type, respectively; Fig. 1b). Among patients with *B2M*^*MUT*^ tumours, 3 out of 21 (14%) individuals experienced a complete response (according to RECIST1.1 criteria), 12 (57%) experienced a partial response, 5 (24%) experienced a durable stable disease and 1 (4.8%) experienced progressive disease as the best overall response. All 44 *B2M* alterations across 21 patients were clonal (Methods), consistent with previous observations in MMR-d cancers^18^. A total of 13 out of 21 (62%) patients with *B2M*^*MUT*^ tumours had biallelic *B2M* alterations, 4 (19%) had potentially biallelic alterations and 4 (19%) had non-biallelic alterations (Fig. 1c and Methods). Non-biallelic alterations have also been associated with complete loss of B2M protein expression in MMR-d tumours^18^. Thus, *B2M* alterations are associated with a high clinical benefit rate of PD-1 blockade in patients with MMR-d cancers.

## Vδ1 and Vδ3 TCRs are overexpressed in *B2M*^*MUT*^ cancers

To gain insights into the immune cell subsets that are involved in immune responses to HLA-class-I-negative MMR-d cancers, we used data of The Cancer Genome Atlas (TCGA) and studied the transcriptomic changes associated with the genomic loss of *B2M* in three cohorts of individuals with MMR-d cancer in colon adenocarcinoma (COAD; *n* = 50 (*B2M*^*WT*^), *n* = 7 (*B2M*^*MUT*^)), stomach adenocarcinoma (STAD; *n* = 48 (*B2M*^*WT*^) and *n* = 12 (*B2M*^*MUT*^)), and endometrium carcinoma (UCEC; *n* = 118 (*B2M*^*WT*^) and *n* = 4 (*B2M*^*MUT*^)). We found that *B2M* was among the most significantly downregulated genes in *B2M*^*MUT*^ cancers (two-sided limma-voom-based regression, *P* = 3.5 × 10^−4^, Benjamini–Hochberg false-discovery rate (FDR)-adjusted *P* = 0.12, adjusted for tumour type; Fig. 1d). Genes encoding components of the HLA class I antigen presentation machinery other than *B2M* were highly upregulated in *B2M*^*MUT*^ tumours, which may reflect reduced evolutionary pressure on somatic inactivation of these genes in the *B2M*^*MUT*^ context^18^ (Fig. 1d). Notably, we found *TRDV1* and *TRDV3*, which encode the variable regions of the δ1 and δ3 chains of the γδ T cell receptor (TCR), among the most significantly upregulated loci in *B2M*^*MUT*^ tumours (*TRDV1*, two-sided limma-voom-based regression, FDR-adjusted *P* = 0.00090, adjusted for tumour type; *TRDV3*, two-sided limma-voom-based regression, FDR-adjusted *P* = 0.0015, adjusted for tumour type; Fig. 1d), regardless of the allelic status of the *B2M* alteration (Extended Data Fig. 1d). Consistent with this, the expression levels of *TRDV1* and *TRDV3* were higher in *B2M*^*MUT*^ compared with in *B2M*^*WT*^ MMR-d cancers (two-sided Wilcoxon rank-sum test, *P* = 6.5 × 10^−8^ for all of the cohorts combined; two-sided linear regression, *P* = 4.7 × 10^−6^, adjusted for tumour type; Fig. 1d–f). Moreover, *B2M*^*MUT*^ tumours showed overexpression of multiple KIRs (Fig. 1d), which clustered together with *TRDV1* and *TRDV3* on the basis of hierarchical clustering (Extended Data Fig. 1e). The expression level of different KIRs (Supplementary Table 2) was higher in *B2M*^*MUT*^ tumours compared with in *B2M*^*WT*^ MMR-d tumours (two-sided Wilcoxon rank-sum test, *P* = 4.4 × 10^−6^ for all cohorts combined; two-sided linear regression, *P* = 4.7 × 10^−5^, adjusted for tumour type; Fig. 1d–f). Together, these results suggest that ICB-naive *B2M*^*MUT*^ MMR-d cancers show increased levels of Vδ1 and Vδ3 T cells as well as increased numbers of these or other immune cells expressing KIRs—a potential mechanism of recognition of HLA class I loss.

We used marker gene sets (modified from ref. ^24^; Methods and Supplementary Table 2) to estimate the abundance of a broad set of other immune cell types on the basis of the RNA expression data of the TCGA cohorts. Hierarchical clustering identified a high- and a low-infiltrated cluster in each of the three tumour types (Fig. 1e). Compared with the Vδ1 and Vδ3 T cell and KIR gene sets, the other marker gene sets showed no or only weak association between expression level and *B2M* status, indicating that our findings were not solely driven by a generally more inflamed state of *B2M*^*MUT*^ tumours (Fig. 1e,f and Extended Data Fig. 1f).

We next revisited the DRUP cohort and specifically applied the marker gene sets to RNA expression data. Despite the low patient numbers and high heterogeneity regarding tumour types and biopsy locations of this cohort, we confirmed increased *TRDV1* and *TRDV3* expression in *B2M*^*MUT*^ tumours pan-cancer (two-sided linear regression, *P* = 0.017, adjusted for tumour type and biopsy site; Fig. 1g, Extended Data Fig. 1g and Methods). KIR expression was significantly associated with *B2M* status only in CRC (Fig. 1g). Across mismatch repair-proficient (MMR-p) metastatic cancers in the Hartwig database^25^, 36 out of 2,256 (1.6%) cancers had a clonal *B2M* alteration, which was frequently accompanied by loss of heterozygosity (LOH) (Extended Data Fig. 1h and Supplementary Table 3). Although rare, *B2M* alterations were also significantly associated with increased expression of *TRDV1*/*TRDV3* loci in this context (two-sided linear regression, *P* = 2.2 × 10^−17^, adjusted for tumour type; Extended Data Fig. 1i and Methods). Taken together, *B2M* defects are positively associated with clinical benefits of ICB treatment, as well as infiltration by Vδ1 and Vδ3 T cells and expression of KIRs.

## Vδ1 and Vδ3 T cells are activated in MMR-d CRC

**Fig. 2:**
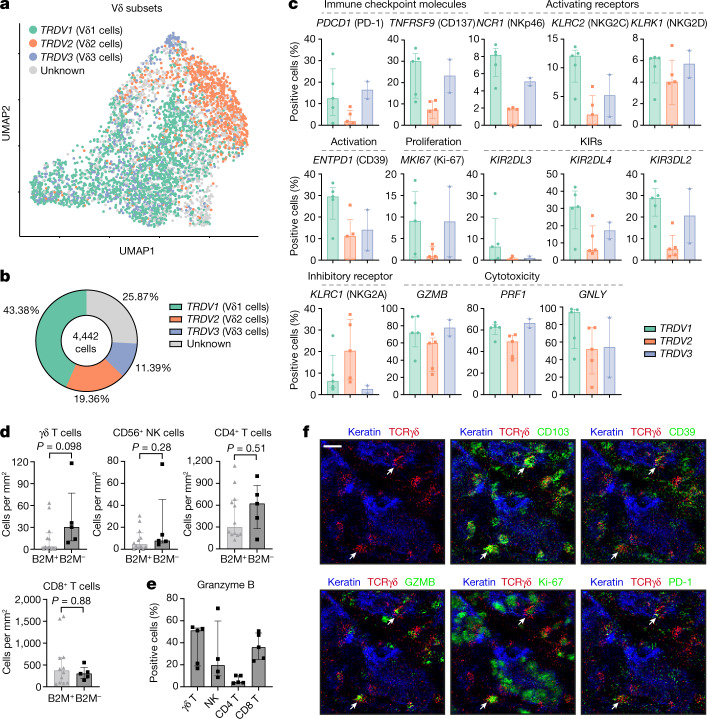
Tumour-infiltrating Vδ1 and Vδ3 T cell subsets display hallmarks of cytotoxic activity in MMR-d colon cancers. **a**, UMAP embedding showing the clustering of γδ T cells (*n* = 4,442) isolated from MMR-d colon cancers (*n* = 5) analysed using scRNA-seq. The colours represent the TCR Vδ chain usage. The functionally distinct γδ T cell clusters are shown in Extended Data Fig. 3. Dots represent single cells. **b**, The frequencies of TCR Vδ chain use of the γδ T cells (*n* = 4,442) analysed using scRNA-seq as a percentage of total γδ T cells. **c**, The frequencies of positive cells for selected genes across Vδ1 (*n* = 1,927), Vδ2 (*n* = 860) and Vδ3 (*n* = 506) cells as the percentage of total γδ T cells from each MMR-d colon tumour (*n* = 5) analysed using scRNA-seq. Vδ3 cells were present in two out of five colon cancers. Data are median ± IQR, with individual samples (dots). **d**, The frequencies of γδ T cells, CD56^+^ NK cells, CD4^+^ T cells and CD8^+^ T cells in treatment-naive B2M^+^ (*n* = 12) and B2M^–^ (*n* = 5) MMR-d colon cancers. Data are median ± IQR, with individual samples (dots). *P* values were calculated using two-sided Wilcoxon rank-sum tests. **e**, The frequencies of granzyme-B-positive γδ T cells, CD56^+^ NK cells, CD4^+^ T cells and CD8^+^ T cells in treatment-naive B2M^–^ (*n* = 5) MMR-d colon cancers. CD56^+^ NK cells were present in four out of five B2M^–^ cancer samples. Data are median ± IQR, with individual samples (dots). **f**, Representative images of the detection of tissue-resident (CD103^+^), activated (CD39^+^), cytotoxic (granzyme B^+^), proliferating (Ki-67^+^) and PD-1^+^ γδ T cells (white arrows) by IMC analysis of a treatment-naive MMR-d colon cancer with B2M defects. Scale bar, 20 μm.

**Extended Data Fig. 2:**
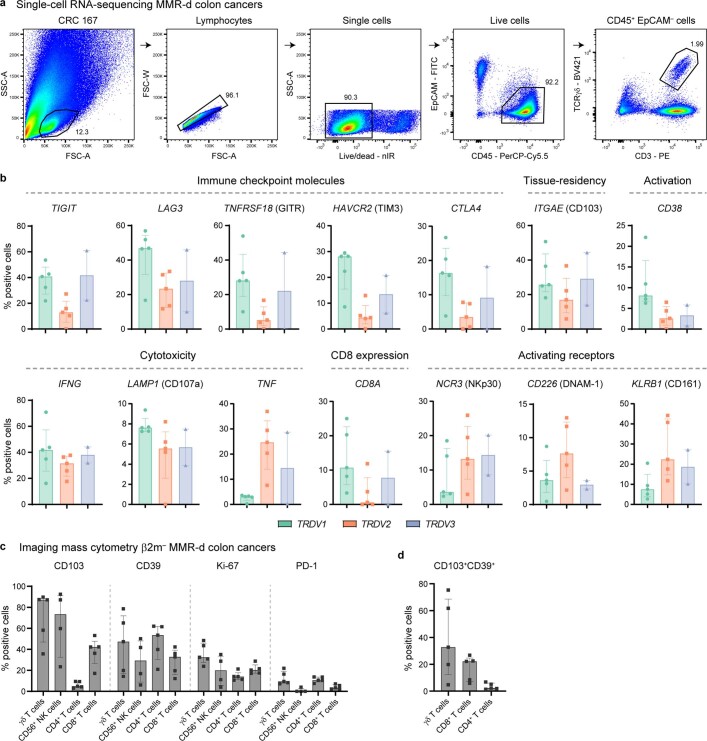
Characterization of γδ T cells and other immune cell populations infiltrating MMR-d colon cancers. **a**. FACS gating strategy for single, live CD45^+^ EpCAM^–^ CD3^+^ TCRγδ^+^ cells from a representative MMR-d colon cancer sample showing sequential gates with percentages. **b**. Frequencies of positive cells for selected genes across Vδ1 (n = 1927), Vδ2 (n = 860), and Vδ3 (n = 506) cells as percentage of total γδ T cells from each MMR-d colon tumour (n = 5) analysed by single-cell RNA-sequencing. Vδ3 cells were present in two out of five colon cancers. Bars and dots indicate median ± IQR and individual samples, respectively. **c**. Frequencies of marker-positive γδ T cells, CD56^+^ NK cells, CD4^+^ T cells, and CD8^+^ T cells in treatment-naive β2m^–^ (n = 5) MMR-d colon cancers. CD56^+^ NK cells were present in four out of five β2m^–^ cancer samples. Bars and dots indicate median ± IQR and individual samples, respectively. **d**. Frequencies of CD103^+^CD39^+^ γδ T cells, CD8^+^ T cells, and CD4^+^ T cells in treatment-naive β2m^–^ (n = 5) MMR-d colon cancers. Bars indicate median ± IQR and individual samples, respectively.

**Extended Data Fig. 3:**
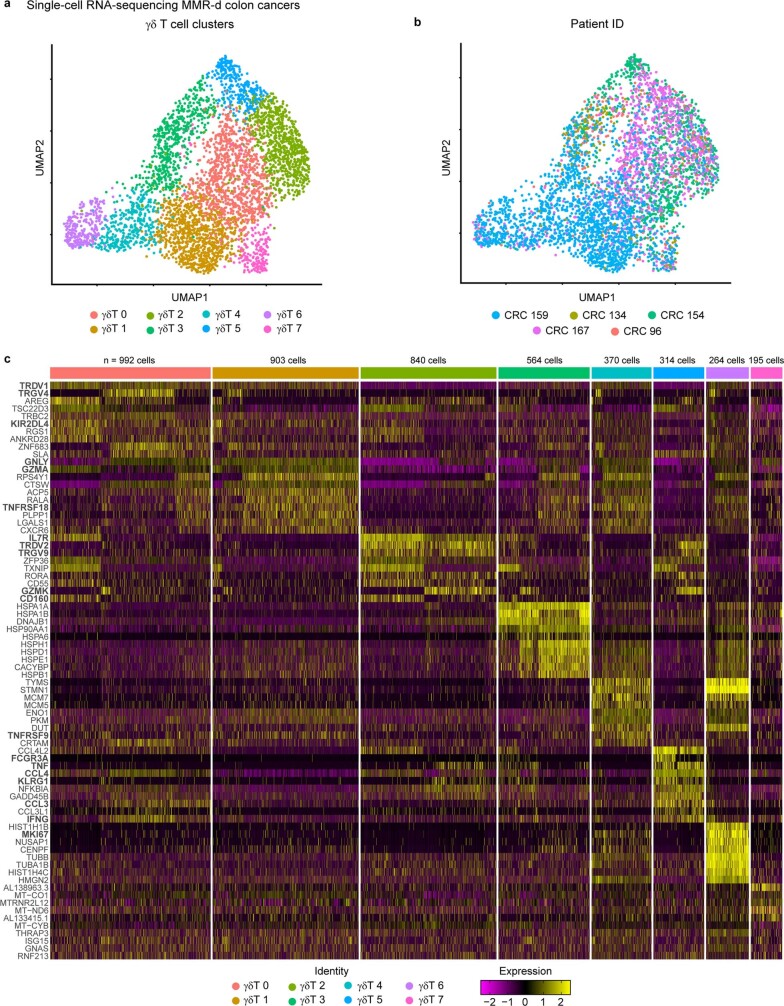
Distinct clusters of γδ T cells in MMR-d colon cancers by single-cell RNA-sequencing. **a**. UMAP embedding showing γδ T cells (n = 4442) isolated from MMR-d colon cancers (n = 5) analysed by single-cell RNA-sequencing. Colours represent the functionally different γδ T cell clusters identified by graph-based clustering and non-linear dimensional reduction. Dots represent single cells. **b**. UMAP embedding of (**a**) coloured by patient ID. Dots represent single cells. **c**. Heatmap showing the normalized single-cell gene expression value (z-score, purple-to-yellow scale) for the top 10 differentially expressed genes in each identified γδ T cell cluster.

To investigate which γδ T cell subsets are present in MMR-d colon cancers and to determine their functional characteristics, we performed single-cell RNA-sequencing (scRNA-seq) analysis of γδ T cells isolated from five MMR-d colon cancers (Extended Data Figs. 2 and 3 and Supplementary Table 4). Three distinct Vδ subsets were identified (Fig. 2a)—Vδ1 T cells were the most prevalent (43% of γδ T cells), followed by Vδ2 (19%) and Vδ3 T cells (11%) (Fig. 2b). *PDCD1* (encoding PD-1) was predominantly expressed by Vδ1 and Vδ3 T cells, whereas Vδ1 cells expressed high levels of genes that encode activation markers such as CD39 (*ENTPD1*) and CD38 (Fig. 2c and Extended Data Fig. 2b). Furthermore, proliferating γδ T cells (expressing *MKI67*) were especially observed in the Vδ1 and Vδ3 subsets (Fig. 2c). Other distinguishing features of the Vδ1 and Vδ3 T cell subsets included the expression of genes encoding activating receptors NKp46 (encoded by *NCR1*), NKG2C (encoded by *KLRC2*) and NKG2D (encoded by *KLRK1*) (Fig. 2c). Notably, the expression of several KIRs was also higher in the Vδ1 and Vδ3 subsets as compared to Vδ2 T cells (Fig. 2c). Almost all γδ T cells displayed expression of the genes encoding granzyme B (*GZMB*), perforin (*PRF1*) and granulysin (*GNLY*) (Fig. 2c). Together, these data support a role for γδ T cells in mediating natural cytotoxic antitumour responses in HLA-class-I-negative MMR-d colon cancers.

Next, we applied imaging mass cytometry (IMC) analysis to a cohort of 17 individuals with ICB-naive MMR-d colon cancers (Supplementary Table 4). High levels of γδ T cell infiltration were observed in cancers with B2M defects as compared to B2M-proficient cancers, albeit this difference was not significant (Fig. 2d). The levels of other immune cells, including NK cells, CD4^+^ T cells and CD8^+^ T cells, were similar between B2M-deficient and B2M-proficient tumours (Fig. 2d). In B2M-deficient cancers, γδ T cells showed frequent intraepithelial localization and expression of CD103 (tissue-residency), CD39 (activation), granzyme B (cytotoxicity) and Ki-67 (proliferation), as well as PD-1 (Fig. 2d–f and Extended Data Fig. 2c), consistent with the scRNA-seq data. Notably, γδ T cells in B2M-deficient cancers showed co-expression of CD103 and CD39 (Extended Data Fig. 2d), which has been reported to identify tumour-reactive CD8^+^ αβ T cells in a variety of cancers^26^.

## PD-1^+^ Vδ1 and Vδ3 T cells kill HLA-class-I^–^ CRC cells

**Fig. 3:**
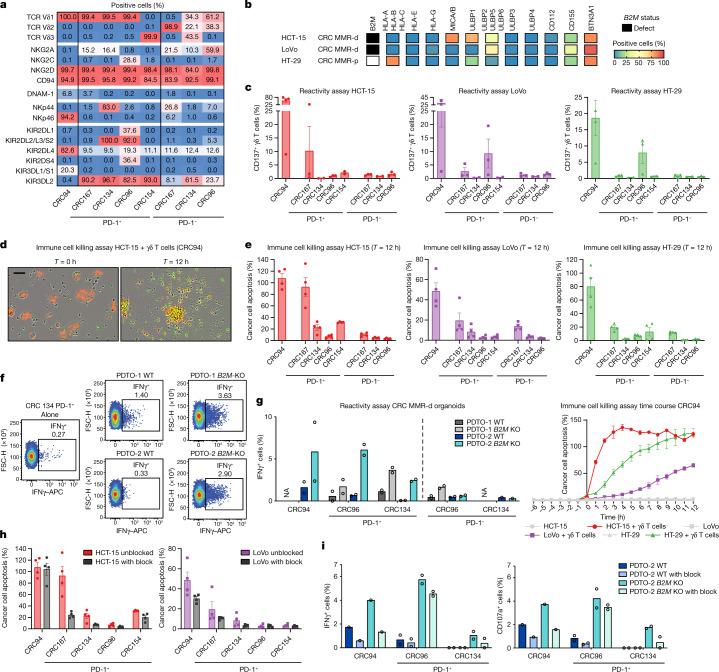
γδ T cells from MMR-d colon cancers show preferential reactivity to HLA-class-I-negative cancer cell lines and organoids. **a**, The percentage of positive cells for the indicated markers on expanded γδ T cells from MMR-d colon cancers (*n* = 5). **b**, Diagram showing the *B2M* status and surface expression of HLA class I, NKG2D ligands, DNAM-1 ligands and butyrophilin on CRC cell lines. MMR-p, MMR proficient. **c**, CD137 expression on γδ T cells after co-culture with CRC cell lines. Data are mean ± s.e.m. from at least two independent experiments. **d**, Representative images showing the killing of NucLightRed-transduced HCT-15 cells by γδ T cells in the presence of a green fluorescent caspase-3/7 reagent. Cancer cell apoptosis is visualized in yellow. Scale bar, 50 μm. **e**, Quantification of the killing of CRC cell lines after co-culture with γδ T cells as described in **d**. Data are mean ± s.e.m. of two wells with two images per well. A representative time course of cancer cell apoptosis is shown at the bottom right. **f**, Representative flow cytometry plots showing IFNγ expression in γδ T cells unstimulated (alone) and after stimulation with two *B2M*^*WT*^ and *B2M*^*KO*^ CRC MMR-d organoids. **g**, IFNγ expression in γδ T cells after stimulation with two *B2M*^*WT*^ and *B2M*^*KO*^ CRC MMR-d organoids, shown as the difference compared with the unstimulated γδ T cell sample. Data are from two biological replicates, except for a single biological replicate of CRC134 PD-1^–^. NA, not available. **h**, The killing of CRC cell lines after 12 h co-culture with γδ T cells with or without NKG2D ligand blocking. Data are mean ± s.e.m. of two wells with two images per well. **i**, IFNγ (left) and CD107a (right) expression in γδ T cells after stimulation with *B2M*^*WT*^ PDTO-2 or *B2M*^*KO*^ PDTO-2, with or without NKG2D ligand blocking and subtracted background signal. Data are from two biological replicates, except for a single biological replicate of CRC94.

**Extended Data Fig. 4:**
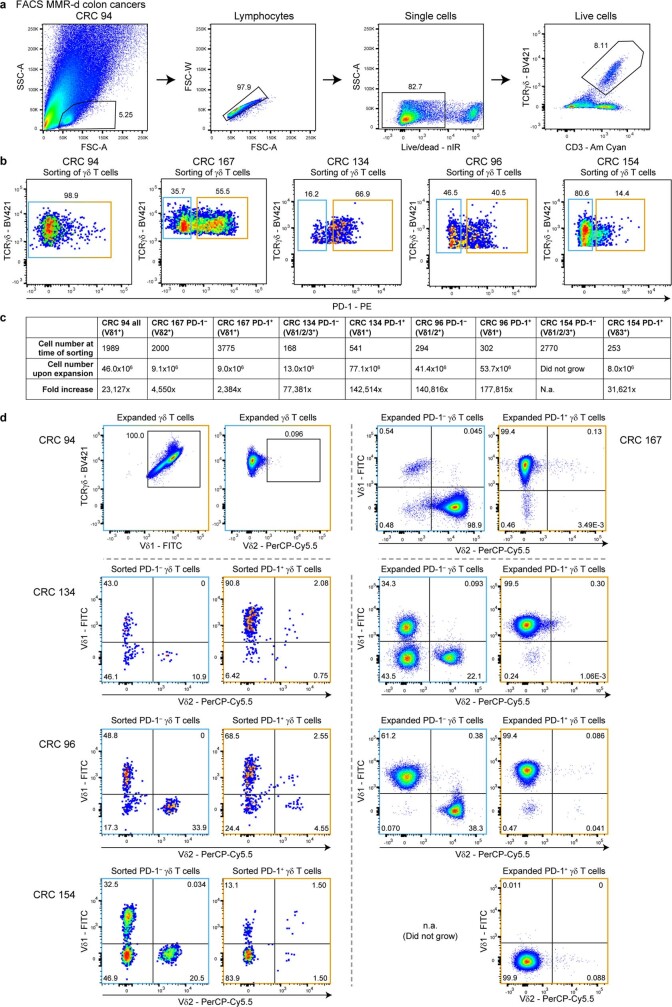
Sorting and expansion of γδ T cells from MMR-d colon cancers. **a**. FACS gating strategy for single, live CD3^+^ TCRγδ^+^ cells from a representative MMR-d colon cancer sample showing sequential gates with percentages. **b**. Sorting of all γδ T cells from CRC94 (due to the low number of PD-1^+^ cells), and of PD-1^–^ (blue squares) and PD-1^+^ (orange squares) γδ T cells from CRC167, CRC134, CRC96, and CRC154. Dots represent single cells. **c**. Table showing the number of γδ T cells isolated from MMR-d colon cancers (n = 5) at the time of sorting *vs* 3-4 weeks after expansion, and the fold increase thereof. **d**. TCR Vδ chain usage after expansion of γδ T cells from CRC94 and CRC167 (first row), and at the time of sorting (left panel) as well as after expansion (right panel) of γδ T cells from CRC134, CRC96, and CRC154. PD-1^–^ γδ T cells from CRC154 did not expand in culture. Dots represent single cells.

**Extended Data Fig. 5:**
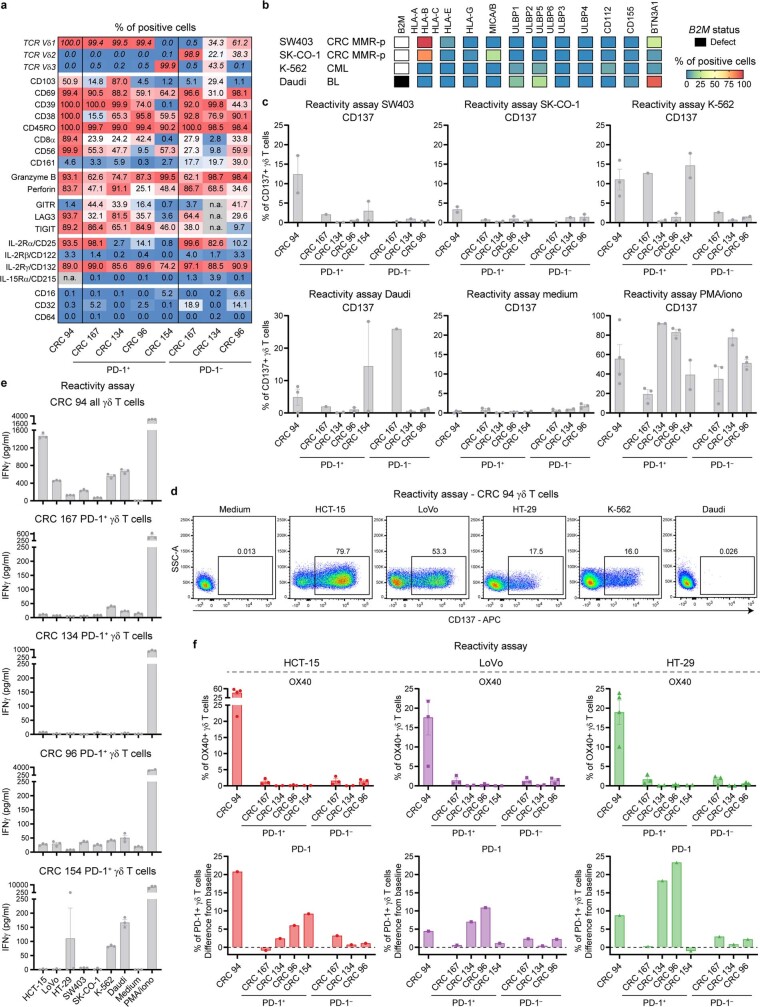
Phenotype and reactivity of γδ T cells towards cancer cell lines. **a**. The percentage of positive cells for the indicated markers on expanded γδ T cells from MMR-d colon cancers (n = 5). **b**. Diagram showing the *B2M* status and surface expression of HLA class I, NKG2D ligands, DNAM-1 ligands, and butyrophilin on cancer cell lines. **c**. Bar plots showing CD137 expression on γδ T cells upon co-culture with cancer cell lines. Medium was used as negative control and PMA/ionomycin as positive control. Bars indicate mean ± SEM. Data from at least two independent experiments except for CRC167, depending on availability of γδ T cells. **d**. Representative flow cytometry plots showing CD137 expression on γδ T cells from a MMR-d colon cancer upon co-culture with cancer cell lines as compared to medium only. Gates indicate percentage of positive γδ T cells. **e**. Bar plots showing the presence of IFNγ in the supernatant upon co-culture of γδ T cells from MMR-d colon cancers (n = 5) with cancer cell lines. Medium as negative control and PMA/ionomycin as positive control are included. Bars indicate mean ± SEM of triplicates. **f**. Bar plots showing the expression of OX40 (first row) and PD-1 (second row) on γδ T cells upon co-culture of γδ T cells with CRC cell lines. PD-1 expression is shown as difference from baseline (medium) condition. Bars indicate mean ± SEM. Data from at least two independent experiments.

We next sought to determine whether tumour-infiltrating γδ T cells can recognize and kill CRC cells. We isolated and expanded PD-1^–^ and PD-1^+^ γδ T cells from five MMR-d colon cancers (Extended Data Fig. 4a–c and Supplementary Table 4). Consistent with the scRNA-seq data, expanded PD-1^+^ γδ T cell populations lacked Vδ2^+^ cells and comprised the Vδ1^+^ or Vδ3^+^ subsets, whereas the PD-1^–^ fractions contained Vδ2^+^ or a mixture of Vδ1^+^, Vδ2^+^ and Vδ3^+^ populations (Fig. 3a and Extended Data Fig. 4d). Detailed immunophenotyping of the expanded γδ T cells (Fig. 3a and Extended Data Fig. 5a) showed that all of the subsets expressed the activating receptor NKG2D, whereas the surface expression of natural cytotoxicity receptors (NCRs) and KIRs was most frequent on PD-1^+^ γδ T cells (Vδ1 or Vδ3^+^), consistent with the scRNA-seq results of unexpanded populations.

**Extended Data Fig. 6:**
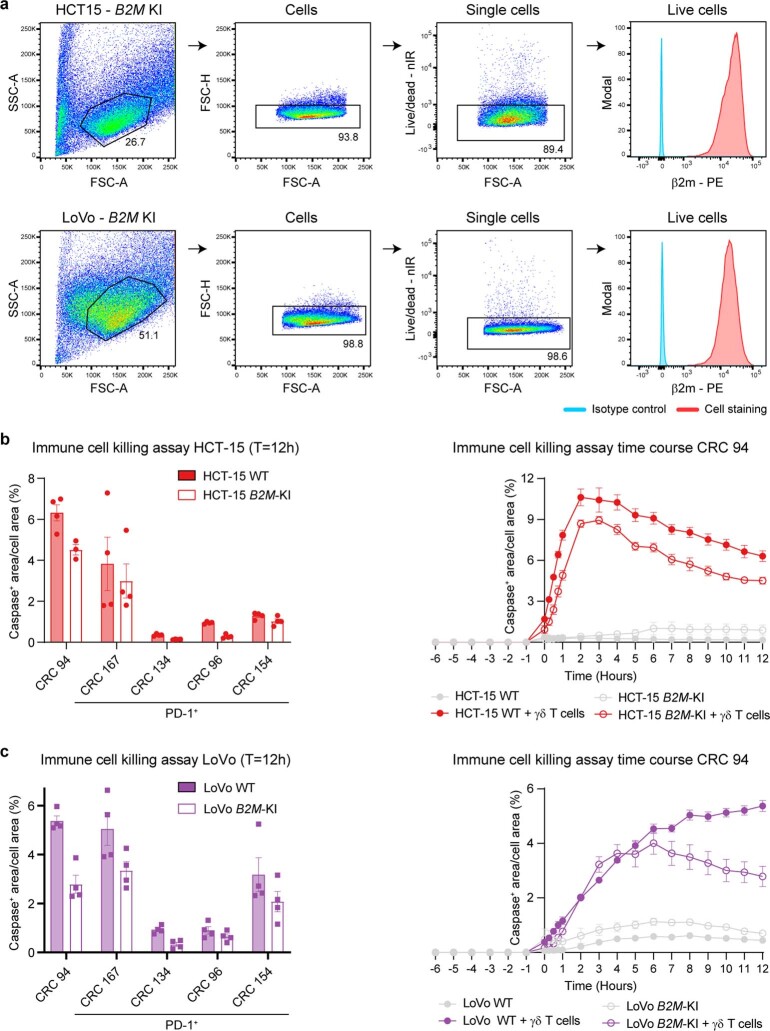
Reactivity of γδ T cells towards *B2M*-knockin vs -wildtype cancer cell lines. **a**. Flow cytometry gating strategy to validate β2m expression on HCT-15 and LoVo *B2M*-knockin (*B2M*-KI) cell lines. Isotype controls were included as negative control. **b**. Bar plots showing the quantification of killing of HCT-15 *B2M*-KI *vs* wildtype (WT) cells upon co-culture with γδ T cells from MMR-d colon cancers (n = 5) in the presence of a red fluorescent caspase-3/7 reagent. Bars indicate mean ± SEM of two wells with two images/well. Right panel shows representative time course of apoptosis. **c**. As **b**, but for LoVo *B2M*-KI *vs* WT cells.

We measured the reactivity of the expanded γδ T cell populations to HLA-class-I-negative and HLA-class-I-positive cancer cell lines (Fig. 3b and Extended Data Fig. 4b). After co-culture with the different cancer cell lines, reactivity (assessed by expression of activation markers and secretion of IFNγ) was largely restricted to PD-1^+^ γδ T cells (Vδ1 or Vδ3^+^), whereas activation of PD-1^–^ γδ T cells (Vδ2^+^) was generally not detected (Fig. 3c and Extended Data Fig. 4). PD-1^+^ γδ T cell (Vδ1 or Vδ3^+^) reactivity was variable and was observed against both HLA-class-I-negative and HLA-class-I-positive cell lines (Fig. 3c and Extended Data Fig. 4). To quantify and visualize the differences in the killing of CRC cell lines by PD-1^+^ and PD-1^–^ γδ T cells, we co-cultured the γδ T cell populations with three CRC cell lines (HCT-15, LoVo, HT-29) in the presence of a fluorescent cleaved-caspase-3/7 reporter to measure cancer cell apoptosis over time (Fig. 3d,e). We found pronounced cancer cell apoptosis after co-culture with PD-1^+^ γδ T cells (Vδ1 or Vδ3^+^) compared with PD-1^–^ cells; cancer cell death was more pronounced in HLA-class-I-negative HCT-15 cells (Fig. 3e and Supplementary Videos 1 and 2). Reintroduction of *B2M* in the *B2M*-deficient HCT-15 and LoVo cells diminished their killing by PD-1^+^ γδ T cells (Vδ1 or Vδ3^+^) cells (Extended Data Fig. 6), suggesting that *B2M* loss increases the sensitivity to γδ T cells.

**Extended Data Fig. 7:**
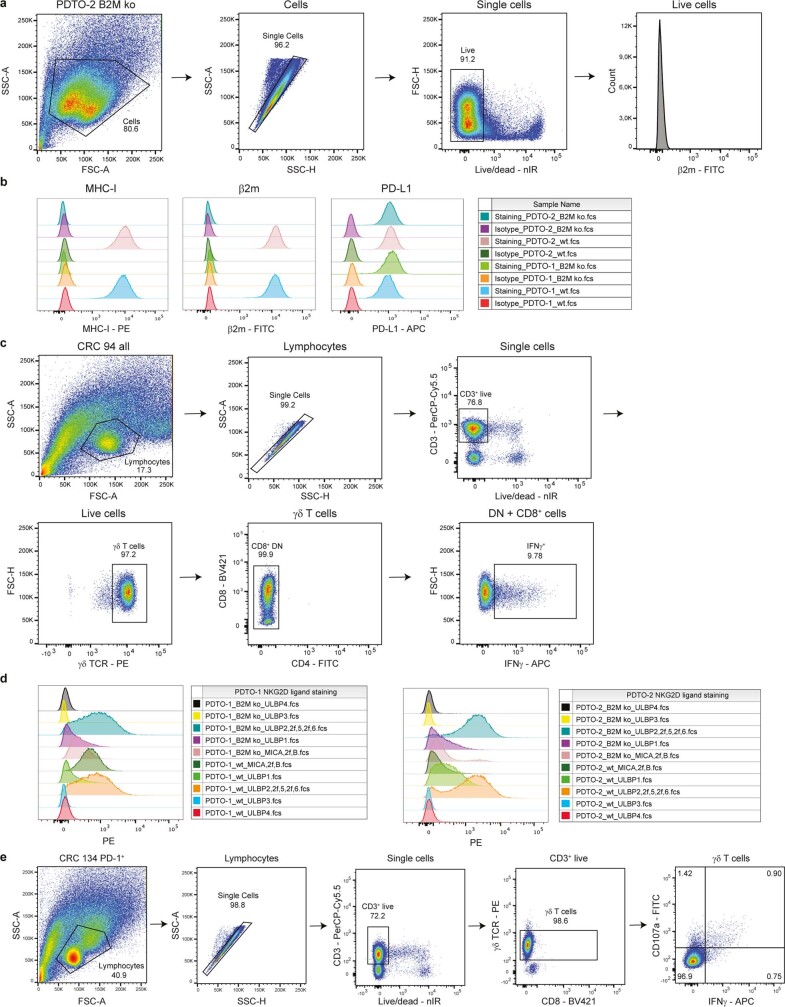
Tumour organoid characterization and reactivity assay readout. **a**. Flow cytometry gating strategy on PDTO cells for analysis of surface staining. Selected cells were gated on single, live cells before quantification of staining signal. **b**. Histogram representation and count for surface staining of MHC-I, PD-L1, and β2m expression on two PDTO lines *B2M*^*WT*^ and *B2M*^*KO*^ after IFNγ pre-stimulation. Staining with isotype antibodies for each fluorochrome (PE, APC and FITC) were included as negative control. **c**. Flow cytometry gating strategy on γδ T cell samples for analysis of intracellular staining to test antitumour reactivity upon PDTO stimulation. Lymphocyte population was further gated on single cells, live and CD3^+^ cells, γδTCR^+^ cells and CD8^+^ as well as CD8^–^CD4^–^ cells. Reactivity of the sample was based on IFNγ^+^ cells of the selected population. **d**. Histogram representation and count for surface staining of NKG2D ligands MICA/B, ULBP1, ULBP2/5/6, ULBP3, and ULBP4 on two PDTO lines *B2M*^*WT*^ and *B2M*^*KO*^ after IFNγ pre-stimulation. **e**. Flow cytometry gating strategy on γδ T cell samples for analysis of intracellular staining after stimulation with PDTOs in the presence of NKG2D ligand blocking. Lymphocyte population was further gated on single cells, live and CD3^+^ cells, followed by γδTCR^+^ and CD8^+^ as well as CD8^–^ cells. Reactivity of final population was based on IFNγ^+^ or CD107a^+^ cells.

Next, we established two parental patient-derived tumour organoid lines (PDTOs; Supplementary Table 5) of MMR-d CRC and generated isogenic *B2M*^*KO*^ lines using CRISPR. Genomic knockout of *B2M* effectively abrogated cell surface expression of HLA class I (Extended Data Fig. 7). We exposed two *B2M*^*KO*^ lines and their parental *B2M*^*WT*^ lines to the expanded γδ T cell subsets, and quantified γδ T cell activation by determination of IFNγ expression. Similar to our cell line data, γδ T cells displayed increased reactivity to *B2M*^*KO*^ PDTOs in comparison to the *B2M*^*WT*^ PDTOs (Fig. 3f,g). Furthermore, γδ T cell reactivity to *B2M*^*KO*^ tumour organoids was preferentially contained within the PD-1^+^ population of γδ T cells (Fig. 3g). Thus, a lack of HLA class I antigen presentation in MMR-d tumour cells can be effectively sensed by γδ T cells and stimulates their antitumour response.

**Extended Data Fig. 8:**
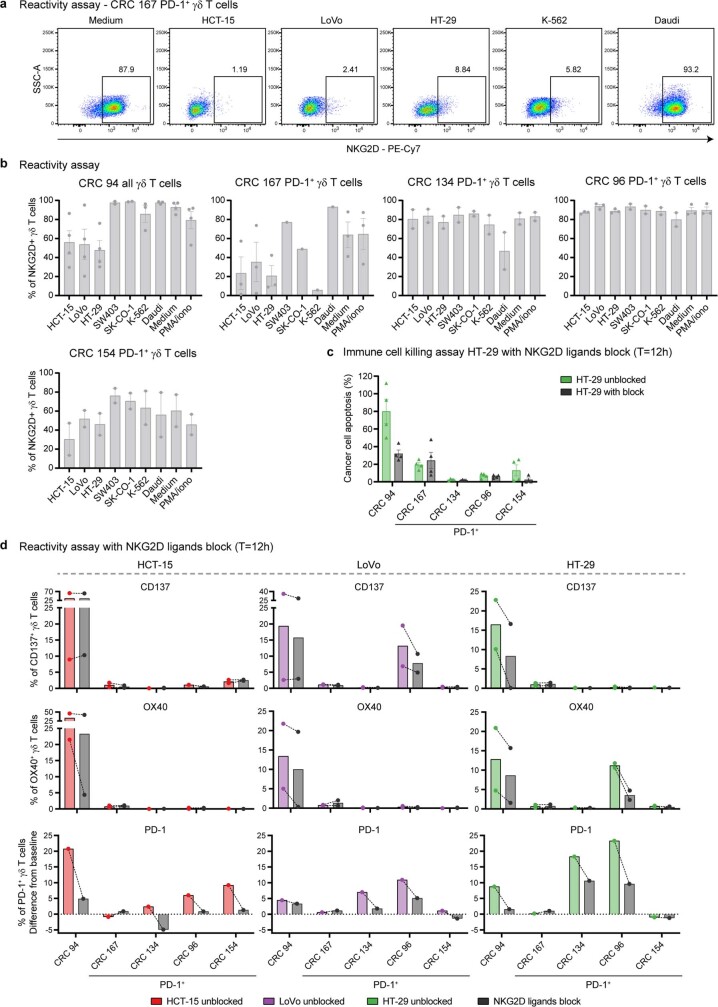
Reactivity of γδ T cells towards cancer cell lines in the presence of NKG2D ligand blocking. **a**. Representative flow cytometry plots showing NKG2D expression on γδ T cells from a MMR-d colon cancer upon co-culture with cancer cell lines as compared to medium only. Gates indicate percentage of positive γδ T cells. **b**. Bar plots showing NKG2D expression on γδ T cells from MMR-d colon cancers (n = 5) upon co-culture with cancer cell lines. Medium as negative control and PMA/ionomycin as positive control are included. Bars indicate mean ± SEM. Data from at least two independent experiments except for CRC167 (SW403, SK-CO-1, K-562), depending on availability of γδ T cells. **c**. Bar plots showing the killing of HT-29 cells upon co-culture with γδ T cells with or without NKG2D ligand blocking. Bars indicate mean ± SEM of two wells with two images/well. **d**. Bar plots showing the expression of CD137 (first row), OX40 (second row), and PD-1 (third row) on γδ T cells upon co-culture of γδ T cells with CRC cell lines with or without NKG2D ligand blocking. PD-1 expression is shown as difference from baseline (medium) condition. Bars and lines indicate mean and similar experiments, respectively. Data from two independent experiments for CD137 and OX40.

Expression of NKG2D on γδ T cells decreased during co-culture with target cells (Extended Data Fig. 8a,b), suggesting the involvement of the NKG2D receptor in γδ T cell activity. The NKG2D ligands MICA/B and ULBPs were expressed by the cancer cell lines (Fig. 3b) and the MMR-d CRC PDTOs, irrespective of their *B2M* status (Extended Data Fig. 7). To examine which receptor–ligand interactions might regulate the activity of PD-1^+^ γδ T cells, we performed blocking experiments focused on (1) NKG2D, (2) DNAM-1 and (3) γδ TCR signalling. Of these candidates, the only consistent inhibitory effect was observed for NKG2D ligand blocking on cancer cells, which decreased the activation and killing ability of most PD-1^+^ γδ T cells (Fig. 3h and Extended Data Fig. 8c,d), confirming the mechanistic involvement of the NKG2D receptor in γδ T cell activation in this context. Moreover, blocking NKG2D ligands on MMR-d CRC PDTOs reduced the PDTO-directed tumour reactivity of γδ T cells from CRC94 and CRC134 (Fig. 3i). Together, these results show that γδ T cell reactivity to MMR-d tumours is partly dependent on NKG2D/NKG2D-ligand interactions.

## ICB boosts Vδ1 and Vδ3 T cells in *B2M*^*MUT*^ CRC

**Fig. 4:**
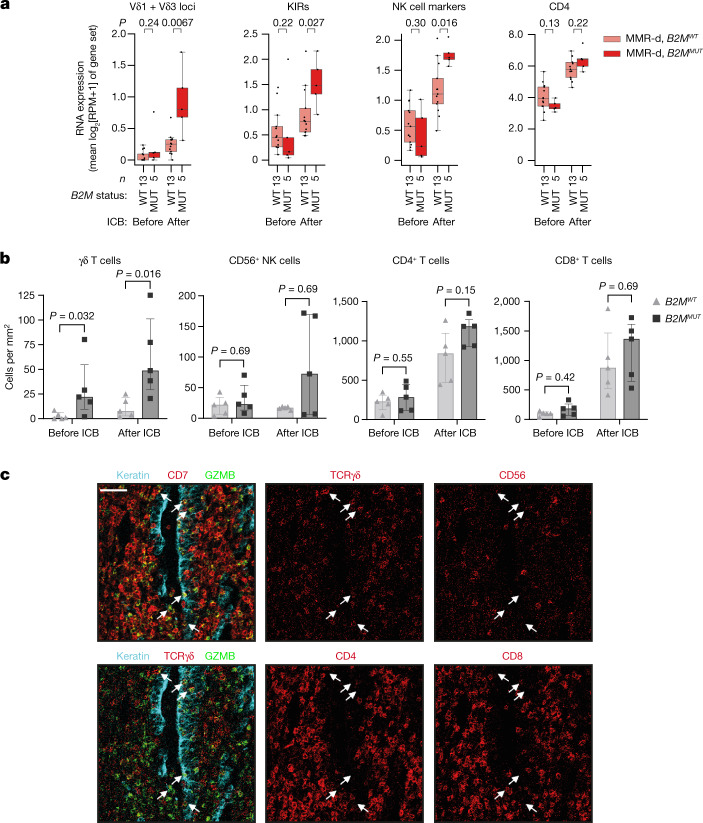
ICB induces substantial infiltration of γδ T cells into MMR-d colon cancers with defects in antigen presentation. **a**, The RNA expression of different immune marker gene sets in MMR-d *B2M*^*WT*^ (pink) and MMR-d *B2M*^*MUT*^ (red) cancers before (left) and after (right) neoadjuvant ICB in the NICHE study. The boxes, whiskers and dots indicate quartiles, 1.5 × IQR and individual data points, respectively. *P* values were calculated using two-sided Wilcoxon rank-sum tests comparing MMR-d *B2M*^*WT*^ versus MMR-d *B2M*^*MUT*^ cancers. **b**, The frequencies of γδ T cells, CD56^+^ NK cells, CD4^+^ T cells and CD8^+^ T cells in *B2M*^*WT*^ (*n* = 5) and *B2M*^*MUT*^ (*n* = 5) MMR-d colon cancers before and after ICB treatment. Data are median ± IQR, with individual samples (dots). *P* values were calculated using two-sided Wilcoxon rank-sum tests. **c**, Representative images of granzyme-B-positive γδ T cells infiltrating the tumour epithelium (white arrows) by IMC analysis of a *B2M*^*MUT*^ MMR-d colon cancer after ICB treatment. Scale bar, 50 μm.

**Extended Data Fig. 9:**
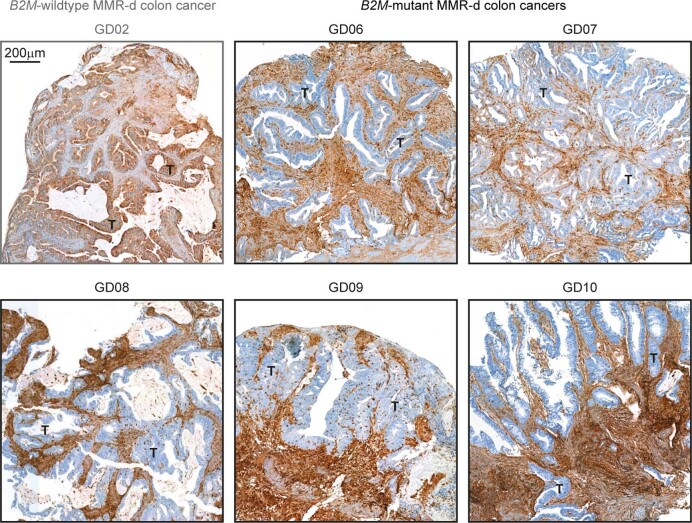
Loss of β2m protein expression on tumour cells in *B2M*-mutant MMR-d colon cancers. Immunohistochemical analysis of β2m protein expression in FFPE tissue from all five *B2M*^*MUT*^ MMR-d colon cancers of the NICHE cohort. A *B2M*^*WT*^ case (GD02) staining positive for β2m is included as control. Details on the staining procedure can be found in Methods.

**Extended Data Fig. 10:**
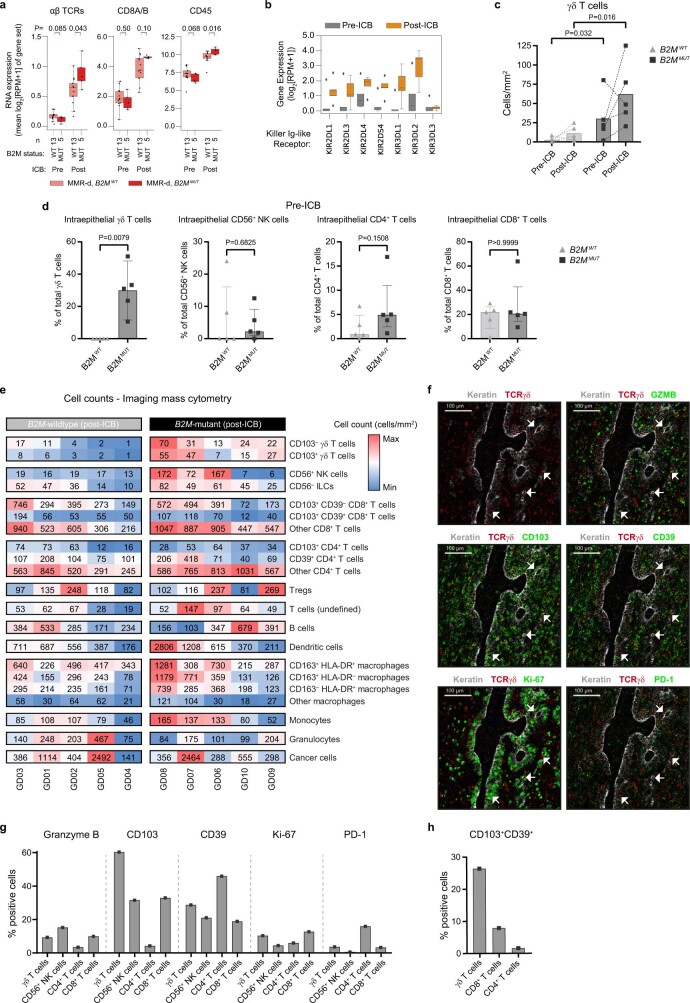
Distribution of immune cell populations in *B2M*-mutant and *B2M*-wildtype MMR-d colon cancers pre- and post-ICB treatment in the NICHE trial. **a**. Immune marker gene set RNA expression in MMR-d *B2M*^*WT*^ (pink), and MMR-d *B2M*^*MUT*^ (red) cancers, before (left) and after (right) neoadjuvant ICB. Boxes, whiskers, and dots indicate quartiles, 1.5 interquartile ranges, and individual data points, respectively. Wilcoxon rank sum test-based two-sided P-values are shown. **b**. Pre- (grey) and post-ICB (orange) RNA expression of KIRs in *B2M*^*MUT*^ MMR-d cancers in the NICHE study (n = 5). Boxes, whiskers, and dots indicate quartiles, 1.5 interquartile ranges, and outliers, respectively. Two-sided P-values were calculated by Wilcoxon rank sum test. **c**. Pre- and post-ICB frequencies of γδ T cells by imaging mass cytometry in *B2M*^*WT*^ (n = 5) and *B2M*^*MUT*^ (n = 5) MMR-d colon cancers (corresponding to Fig. 4b). Lines indicate paired samples, dots represent individual samples. Wilcoxon rank sum test-based two-sided P-values are shown. **d**. Imaging mass cytometry-based frequencies of intraepithelial γδ T cells, CD56^+^ NK cells, CD4^+^ T cells, and CD8^+^ T cells in ICB-naive *B2M*^*WT*^ (n = 5) and *B2M*^*MUT*^ (n = 5) MMR-d colon cancers. Bars and dots indicate median ± IQR and individual samples, respectively. Wilcoxon rank sum test-based two-sided P-values are shown. **e**. Cell counts (cells/mm^2^) of immune populations from the imaging mass cytometry of *B2M*^*WT*^ (n = 5) and *B2M*^*MUT*^ (n = 5) MMR-d colon cancers upon ICB treatment. Colour bar is scaled per immune population. **f**. Representative images of the detection of cytotoxic (granzyme B^+^), tissue-resident (CD103^+^), activated (CD39^+^), proliferating (Ki-67^+^), and PD-1^+^ γδ T cells by imaging mass cytometry in a *B2M*^*MUT*^ MMR-d colon cancer upon ICB treatment. **g**. Frequencies of marker-positive γδ T cells, CD56^+^ NK cells, CD4^+^ T cells, and CD8^+^ T cells in the sole *B2M*^*MUT*^ MMR-d colon cancer that contained cancer cells upon ICB treatment. **h**. Frequencies of CD103^+^CD39^+^ γδ T cells, CD8^+^ T cells, and CD4^+^ T cells in the sole *B2M*^*MUT*^ MMR-d colon cancer that contained cancer cells upon ICB treatment.

We subsequently studied how ICB influences γδ T cell infiltration and activation in MMR-d colon cancers in the therapeutic context. For this purpose, we analysed pre- and post-treatment samples of the NICHE trial^9^, in which patients with colon cancer were treated with neoadjuvant PD-1 plus CTLA-4 blockade. Consistent with our observations in the DRUP cohort, 4 out of 5 (80%) individuals with *B2M*^*MUT*^ cancers in the NICHE trial showed a complete pathologic clinical response. Immunohistochemical analysis confirmed the loss of B2M protein expression on tumour cells in all mutated cases (Extended Data Fig. 9). Whereas expression of immune marker gene sets in the pretreatment samples was similar between 5 *B2M*^*MUT*^ versus 13 *B2M*^*WT*^ cancers, ICB induced a clear immunological divergence between these two groups (Fig. 4a). The *B2M*^*MUT*^ subgroup was most significantly associated with higher post-treatment expression of *TRDV1* and *TRDV3* (two-sided Wilcoxon rank-sum test, *P* = 0.0067; Fig. 4a), followed by higher expression of the general immune cell marker CD45, NK-cell-related markers, KIRs and αβTCRs (two-sided Wilcoxon rank-sum test, *P* = 0.016, *P* = 0.016, *P* = 0.027 and *P* = 0.043, respectively; Fig. 4a and Extended Data Fig. 10a). The set of KIRs upregulated after ICB in *B2M*^*MUT*^ cancers (Extended Data Fig. 10b) was consistent with the sets of KIRs upregulated in *B2M*^*MUT*^ MMR-d cancers in TCGA (Fig. 1e), and those expressed by MMR-d tumour-infiltrating γδ T cells (Fig. 2c). Pre- and post-ICB gene expression levels related to CD4 and CD8 infiltration were not associated with *B2M* status (Fig. 4a and Extended Data Fig. 10a).

To quantify and investigate the differences in immune profiles after ICB treatment, we used IMC to analyse tissues derived from five *B2M*^*MUT*^ HLA-class-I-negative and five *B2M*^*WT*^ HLA-class-I-positive cancers before and after ICB treatment. In the ICB-naive setting, *B2M*^*MUT*^ MMR-d colon cancers showed higher γδ T cell infiltration compared with *B2M*^*WT*^ MMR-d colon cancers (two-sided Wilcoxon rank-sum test, *P* = 0.032; Fig. 4b and Extended Data Fig. 10c). Importantly, a large proportion of these γδ T cells showed an intraepithelial localization in *B2M*^*MUT*^ MMR-d colon cancers compared with the *B2M*^*WT*^ samples (two-sided Wilcoxon rank-sum test, *P* = 0.0079; Extended Data Fig. 10d). No significant differences were observed in the infiltration of other immune cells, such as NK cells, CD4^+^ T cells and CD8^+^ T cells, in ICB-naive *B2M*^*MUT*^ versus *B2M*^*WT*^ MMR-d colon cancers (Fig. 4b). ICB treatment resulted in major pathologic clinical responses, and residual cancer cells were absent in most post-ICB samples. All post-ICB tissues showed a profound infiltration of different types of immune cells (Extended Data Fig. 10e), of which γδ T cells were the only immune subset that was significantly higher in ICB-treated *B2M*^*MUT*^ compared with *B2M*^*WT*^ MMR-d colon cancers (two-sided Wilcoxon rank-sum test, *P* = 0.016; Fig. 4b and Extended Data Fig. 10c). In the sole *B2M*^*MUT*^ case that still contained cancer cells after treatment with ICB, the majority of granzyme B^+^ immune cells infiltrating the tumour epithelium were γδ T cells (Fig. 4c). These γδ T cells displayed co-expression of CD103, CD39, Ki-67 and PD-1 (Extended Data Fig. 10f–h). Taken together, these results show that ICB treatment of MMR-d colon cancer increases the presence of activated, cytotoxic and proliferating γδ T cells at the tumour site, especially when these cancers are B2M-deficient, highlighting γδ T cells as effectors of ICB treatment within this context.

## Discussion

CD8^+^ αβ T cells are major effectors of ICB^11,12,27^ and rely on HLA class I antigen presentation of target cells. We confirm and shed light on the paradox that patients with HLA class I defects in MMR-d cancers retain the clinical benefit of ICB, suggesting that other immune effector cells are involved in compensating for the lack of conventional CD8^+^ T cell immunity in this setting. We show that genomic inactivation of *B2M* in MMR-d colon cancers was associated with: (1) an elevated frequency of activated γδ T cells in ICB-naive tumours; (2) an increased presence of tumour-infiltrating γδ T cells after ICB treatment; (3) in vitro activation of tumour-infiltrating γδ T cells by CRC cell lines and PDTOs; and (4) killing of tumour cell lines by γδ T cells, in particular by Vδ1 and Vδ3 subsets expressing PD-1.

Different subsets of γδ T cells exhibit substantially diverse functions that, in the context of cancer, range from tumour-promoting to tumouricidal effects^20,28,29^. Thus, it is of interest to determine what defines antitumour reactivity of γδ T cells. Here we isolated Vδ1/3-expressing PD-1^+^ T cells as well as Vδ2-expressing PD-1^–^ T cells from MMR-d tumour tissues. Our data suggest that especially tumour-infiltrating Vδ1 and Vδ3 T cells can recognize and kill HLA-class-I-negative MMR-d tumours, whereas Vγ9Vδ2 cells, the most studied and main subset of γδ T cells in the blood, appear to be less relevant within this context. This is consistent with other studies showing that the cytotoxic ability of Vδ1 cells generally outperforms their Vδ2 counterparts^30–34^. Notably, reports of the cytotoxicity of tumour-infiltrating Vδ3 cells have been lacking. Furthermore, the observation that PD-1^+^ γδ T cells (Vδ1 and Vδ3 phenotype) demonstrated clearly higher levels of antitumour reactivity compared with their PD-1^–^ counterparts (Vδ2 phenotype) suggests that, as for CD8^+^ αβ T cells^35^, PD-1 expression may be a marker of antitumour reactivity in γδ T cells.

The mechanisms of activation of γδ T cells are notoriously complex and diverse^20^. Specifically, for Vδ1^+^ cells, NKG2D has been described to be involved in tumour recognition, which is dependent on tumour cell expression of NKG2D ligands MICA/B and ULBPs^36–38^. Here, MICA/B and ULBPs were highly expressed by the MMR-d CRC cell lines and tumour organoids, and blocking these ligands reduced γδ T cell activation and cytotoxicity. This suggests a role for the activating receptor NKG2D in γδ T cell immunity to MMR-d tumours. Future research should address the outstanding question of how γδ T cells accumulate in B2M-deficient tumours, and whether the lack of CD8^+^ T cell activity might contribute to the establishment of an attractive niche for γδ T cells and other immune effector cells. Potential mechanisms for the recognition of HLA-class-I-negative phenotypes may include KIR-, NKG2A- and LILRB1-mediated interactions with target cancer cells. Notably, we found that the expression of KIRs was most pronounced on PD-1^+^ γδ T cells (Vδ1 or Vδ3^+^ subsets), which demonstrated anti-tumour activity. Whether the lack of KIR-mediated signalling promotes the survival of γδ T cells and their intratumoural proliferation remains to be studied.

Our findings have broad implications for cancer immunotherapy. First, our findings strengthen the rationale for combining PD-1 blockade with immunotherapeutic approaches to further enhance γδ T-cell-based antitumour immunity. Second, the presence or absence in tumours of specific γδ T cell subsets (such as Vδ1 or Vδ3) may help to define patients who are responsive or unresponsive to ICB, respectively, especially in the case of MMR-d cancers and other malignancies with frequent HLA class I defects, such as stomach adenocarcinoma^39^ and Hodgkin’s lymphoma^40^. Third, our results suggest that MMR-d cancers and other tumours with HLA class I defects may be particularly attractive targets for Vδ1 or Vδ3 T-cell-based cellular therapies.

Although we have provided detailed and multidimensional analyses, it is probable that γδ T cells are not the only factor driving ICB responses in HLA-class-I-negative MMR-d CRC tumours. In this context, other HLA-class-I-independent immune subsets, such as NK cells and neoantigen-specific CD4^+^ T cells may also contribute. The latter were shown to have an important role in the response to ICB (as reported in mouse *B2M*-deficient MMR-d cancer models^41^), and may also support γδ T-cell-driven responses. Notably, no subset equivalent to Vδ1 or Vδ3 T cells has been identified in mice, which complicates their investigation in in vivo models. In conclusion, our results provide strong evidence that γδ T cells are cytotoxic effector cells of ICB treatment in HLA-class-I-negative MMR-d colon cancers, with implications for further exploitation of γδ T cells in cancer immunotherapy.

## Methods

### TCGA data

RNA expression data (raw counts) of the colon adenocarcinoma (COAD), stomach adenocarcinoma (STAD) and Uterus Corpus Endometrium Carcinoma (UCEC) cohorts of The Cancer Genome Atlas (TCGA) Research Network were downloaded through the GDC data portal (https://portal.gdc.cancer.gov) on 10 April 2019. Of these cohorts, mutation, copy number, purity and ploidy data were downloaded from the GDC on 11 November 2021, as the controlled access ABSOLUTE-annotated^42^ MAF file (mutations), SNP6 white-listed copy number segments file (copy numbers) and ABSOLUTE purity/ploidy file of the TCGA PanCanAtlas project^43^. Mismatch-repair-deficiency status was obtained from ref. ^44^ (TCGA subtype = GI.HM-indel or UCEC.MSI).

### DRUP data

A detailed description of the DRUP, including details on patient accrual, study design, oversight and end points was published previously^23^. In brief, the DRUP is a national, non-randomized multidrug and multitumour study in the Netherlands, in which patients receive off-label drugs registered for other treatment indications. These patients had advanced or metastatic solid tumours and had exhausted standard-treatment options, they were required to be at least 18 years of age, with acceptable organ function and performance status (Eastern Cooperative Oncology Group (ECOG) score ≤ 2), and have an objectively evaluable disease of which a fresh baseline tumour biopsy could safely be obtained. We analysed 71 patients with MMR-d cancers recruited and treated with PD-1 blockade in 22 Dutch hospitals participating in the DRUP^23^ between 2016 and 2021. Patients included in this analysis had (1) a clinical follow-up ≥16 weeks after start of PD-1 blockade treatment; (2) WGS data passing standard quality controls (as defined previously, including a sequencing-based tumour purity of ≥20%)^25^; and (3) available RNA-seq data (Supplementary Table 1). MMR-d status was determined using routine diagnostics at the hospital of patient accrual and was confirmed by WGS, on the basis of an MSIseq (v.1.0.0)^45^ score of >4, which represents a predefined threshold^25^. Consistent with the study protocol of the DRUP^23^, the primary outcome measure for our analysis was clinical benefit, defined as disease control of ≥16 weeks, and the secondary outcome measure was best overall response, all assessed according to the RECIST 1.1 guidelines by the local treatment team at the site of accrual. As determined in the study protocol, these outcome measures were considered to be evaluable in patients who received at least two cycles of intravenous study medication, and for whom the response was radiologically or clinically evaluable (at the treating physician’s discretion). For genomics and transcriptomics analyses, fresh frozen tumour biopsies were obtained at the baseline (that is, before PD-1 blockade). WGS analysis (median depths, ~100× and ~40× for tumour and normal, respectively) and bioinformatics analyses were performed as previously described^23,25^, with an optimized pipeline based on open-source tools that is freely available at GitHub (https://github.com/hartwigmedical/pipeline5). The TMB per Mb was determined by counting the genome-wide number of mutations (SNVs, MNVs and indels) and dividing this number by the number of megabases sequenced. For RNA-seq analysis, we extracted total RNA using the QIAGEN QIAsymphony RNA kit (931636). Samples with approximately 100 ng total RNA were prepared using KAPA RNA Hyper + RiboErase HMR (8098131702) and the RNA libraries were paired-end sequenced on the Illumina NextSeq550 platform (2 × 75 bp) or the Illumina NovaSeq6000 platform (2 × 150 bp). Raw RNA reads (FASTA files) were aligned to the human reference genome (GRCh38) using STAR^46^, (v.2.7.7a) using the default settings in two-pass mode.

### Hartwig data

We analysed 2,256 metastatic tumours included in the freely available Hartwig database^25^ that (1) were MMR-p (WGS-based MSIseq^45^ score ≤4); (2) had available WGS data passing standard quality controls (as defined before, including a sequencing-based tumour purity ≥20%)^25^; (3) and had available RNA-seq data. We excluded 89 tumours from rare primary tumour locations, defined as locations with less that <20 patients in our selection. When individual patients had data available of biopsies obtained at different timepoints, we included data of only the first biopsy. Sequencing and bioinformatics were performed identically to the procedures used for the generation of the DRUP dataset (see above). Details of this cohort are provided in Supplementary Table 3.

### NICHE study data

Raw RNA reads (FASTA files) of our recently published NICHE study^9^ (ClinicalTrials.gov: NCT03026140) were generated as described in the original publication and aligned to the human reference genome (GRCh38) with STAR^46^ (v.2.7.7a) using the default settings in two-pass mode. For gene expression quantification, we used the gencode.v35.annotation.gtf annotation file. Somatic mutation data were obtained from DNA-seq of pretreatment tumour biopsies and matched germline DNA, as described in the original publication^9^.

### *B2M* status

Consistent with the notion that both biallelic and monoallelic non-synonymous *B2M* mutations are strongly associated with tumour-specific loss of B2M protein expression^18^, we considered all tumours with at least one somatic, non-synonymous *B2M* mutation to be a *B2M* mutant. As none of the *B2M* mutant tumours in TCGA had *B2M* copy number gains or losses, LOH of *B2M* could easily be assessed by a simple calculation estimating the mutation’s copy number:

$${{\rm{Mut}}}_{{\rm{CN}}}={\rm{round}}\left(2\times \frac{{\rm{VAF}}}{{\rm{purity}}}\right)$$

where Mut_CN_ represents the estimated mutation’s copy number (rounded to an integer value), VAF represents the variant allele frequency of the mutation and purity equals the ABSOLUTE-based^42^ tumour cell fraction of the sample.

A Mut_CN_ equal to 2 was considered to be consistent with LOH, as the most parsimonious explanation of such a result is the scenario in which all tumour-derived reads spanning the region of the *B2M* mutation contain the mutation and none of the tumour-derived reads are WT.

In analyses of patients in the DRUP and Hartwig datasets, LOH of *B2M* mutations was determined as an integrated functionality of PURPLE (v.2.34)^47^. When multiple *B2M* mutations were present within a sample, we manually phased the mutations through inspection of the *B2M*-aligned reads using the Integrative Genomics Viewer (IGV)^48^. Here mutations were phased in case single reads were observed spanning the genomic locations of both mutations. We divided patients with multiple *B2M* mutations into three subgroups: (1) Biallelic, if (a) the multiple mutations were in *trans* AND the integer sum of the mutation copy numbers equalled (or exceeded) the integer copy number of the *B2M* gene (for mutations in *cis*, only one of these mutations was considered in the calculation); or (b) at least one of the mutations showed LOH. (2) Potentially biallelic, if the multiple mutations affected genomic locations too distant to be phased and the (integer) sum of the mutation copy numbers equalled (or exceeded) the integer copy number of the *B2M* gene (for mutations in *cis*, only one of these mutations was considered in the calculation) and none of the mutations showed LOH. (3) Not bi-allelic, if the integer sum of the mutation copy numbers was smaller than the integer copy number of the *B2M* gene (for mutations in *cis*, only one of these mutations was considered in the calculation) and none of the mutations showed LOH.

In these analyses, mutations were considered to be subclonal in the case in which the probability of subclonality was >0.5 (the situation in which a mutation is more likely subclonal than clonal), as determined using PURPLE (v.2.34)^47^.

### Association of *B2M* status with outcome and tumour characteristics

To test whether somatic *B2M* alterations were associated with the clinical benefit rate of patients with MMR-d tumours treated with ICB in the DRUP, we used a Fisher’s exact test (using the Python package Scipy^49^ (v.1.3.1)) for unadjusted analyses and logistic regression (as implemented by the Python package Statsmodels (https://pypi.org/project/statsmodels/; v.0.10.1) for analyses adjusted for the continuous TMB per Mb and/or the primary site of the tumour. The association of *B2M* status with TMB was tested using Scipy’s Wilcoxon rank-sum test. Associations of *B2M* status with the primary site of the tumour or the biopsy location were tested using Scipy’s Fisher’s exact test.

### Association of TMB with ICB treatment outcome

For the DRUP cohort, the association of clinical benefit with TMB was tested using Statsmodels’ Wilcoxon rank-sum test.

### Differential gene expression analysis

Differential RNA expression of genes was tested in R using EdgeR^50^ (v.3.28.1) and Limma^51^ (including Voom^52^) (v.3.42.2). Raw read counts were filtered by removing low-expressed genes. Normalization factors were calculated using EdgeR to transform the raw counts to log_2_[counts per million reads (CPM)] and calculate residuals using Voom. Voom was then used to fit a smoothened curve to the √(residual standard deviation) by average gene expression, which was then plotted for visual inspection to confirm that the appropriate threshold was used for filtering of low-expressed genes (defined as the minimal amount of filtering necessary to overcome a dipping mean-variance trend at low counts). Next, Limma was used to calculate the differential expression of genes on the basis of a linear model fit, considering the smoothened curve for sample weights, and empirical Bayes smoothing of standard errors. FDR-adjusted *P* values were calculated using Benjamini–Hochberg correction of the obtained *P* values.

#### TCGA

Using TCGA data, we calculated the differential expression between tumours with and without mutations in *B2M*, adjusting for tumour type, using the following design formula: expression ∝ Primary_Site + B2M_status (+ intercept by default), for which Primary_Site was a three-levelled factor (COAD, STAD, or UCEC) and B2M_status was a two-levelled factor (mutated, or wildtype).

#### NICHE study

Using NICHE study data, we calculated differential expression between pre- and post-ICB treatment. To respect the paired nature of these data, we used the following design formula: expression ∝ Patient + ICB + intercept, where Patient is a factor for each individual patient and ICB is a two-levelled factor (ICB-treated, yes/no).

### Immune marker gene set expression analysis

To use RNA-seq data to obtain a relative estimate of the infiltration of specific immune cell types within tumours, we calculated the average log_2_[RPM + 1] expression of marker genes that are specifically expressed in the immune cell types of interest. To this end, we used the previously published marker gene sets^24^, and extended this by (1) *CD4* as a CD4^+^ T cell marker gene; (2) *TRDV1* and *TRDV3* as γδ1/3T cell marker genes; and (3) a killer-cell Ig-like receptor (KIR) gene set (comprising all genes of which the name starts with KIR and of which the name contains DL or DS). We excluded the gene set ‘NK CD56^dim^ cells’ of ref. ^24^ (comprising *IL21R*, *KIR2DL3*, *KIR3DL1* and *KIR3DL2*) from our analyses, as three out of four genes within this set were KIRs and this set therefore showed high collinearity/redundancy to our full KIR gene set. As *XLC1* and *XLC2* are highly expressed by tumour-infiltrating γδ T cells, these genes were removed from the NK cell marker gene set and replaced by *KLRF1*, which encodes the well-established NK cell marker NKp80. The resulting gene set consisted of *NCR1* and *KLRF1*, encoding the well-established NK cell markers NKp46 and NKp80, respectively. Finally, we reduced the ‘cytotoxic cells’ marker gene set of ref. ^24^ to those genes in the set encoding cytotoxic molecules (*GZMA*, *GZMB*, *GZMH*, *PRF1*, *GNLY*, *CTSW*). A list of the final collection of our marker gene sets is provided in Supplementary Table 2.

Association of immune cell marker gene set expression with *B2M* alteration status (alteration yes/no) was calculated as follows:

1. For TCGA-study-based analyses, we used (i) the Wilcoxon rank-sum test (for unadjusted analyses) and (ii) ordinary least squares linear regression (for analyses adjusted for tumour type; as implemented by the Python package ‘Statsmodels), using a similar design formula as for the differential gene expression analysis.
2. For DRUP-cohort-based analyses, we used a linear mixed effects model (as implemented by the lmer function of the R package Lme4 (v.1.1.26)), adjusting for tumour type and biopsy site as random effects, using the following design formula: expression ∝ B2M_status + (1|tumour_type) + (1|biopsy_site) + intercept. In subgroup analysis of CRC, we omitted tumour type in this formula: expression ∝ B2M_status + (1|biopsy_site) + intercept.
3. For Hartwig data-based analyses, we used ordinary least squares linear regression (as implemented by the Python package Statsmodels) adjusting for tumour type, using the following design formula: expression ∝ Primary_Site + B2M_status + intercept.
4. For NICHE-study-based analyses, we used the Wilcoxon rank-sum test (as implemented by the Python package Scipy).

### Hierarchical clustering

Hierarchical clustering of expression profiles of individual genes or immune marker gene sets of TCGA cohorts was performed on *Z*-score-transformed log_2_[RPM + 1] expression values, using the Python package Scipy^49^, with Euclidean distance as the distance metric and using the Ward variance minimization algorithm. Here, we used the default settings with one exception—for visualization purposes, the colour threshold was halved in the TCGA-based clustering of individual genes.

### Patient samples

The DRUP study and the generation of the Hartwig database were initiated and conducted on behalf of the Center for Personalized Cancer Treatment (CPCT; ClinicalTrials.gov: NCT02925234, NCT01855477). These studies were approved by the Medical Ethical Committee of the Netherlands Cancer Institute in Amsterdam and the University Medical Center Utrecht, respectively, and were conducted in accordance with good clinical practice guidelines and the Declaration of Helsinki’s ethical principles for medical research. Written informed consent was obtained from all of the study participants. Moreover, primary colon cancer tissues from a total of 17 patients with colon cancer who underwent surgical resection of their tumour at the Leiden University Medical Center (LUMC, The Netherlands; Supplementary Table 4) were used for scRNA-seq, IMC and functional assays. No patient with a previous history of inflammatory bowel disease was included. This study was approved by the Medical Ethical Committee of the Leiden University Medical Center (protocol P15.282), and patients provided written informed consent. Finally, primary colon cancer tissues from ten patients with colon cancer included in the NICHE study (NCT03026140)^9^ carried out at the Netherlands Cancer Institute (NKI, The Netherlands) were used for this study. All samples were anonymized and handled according to the ethical guidelines described in the Code for Proper Secondary Use of Human Tissue in the Netherlands of the Dutch Federation of Medical Scientific Societies.

### Processing of colon cancer tissues

Details on the processing of colon cancer tissues have been described previously^22^. In brief, macroscopic sectioning from the lumen to the most invasive area of the tumour was performed. Tissues were collected in IMDM + GlutaMax medium (Gibco) complemented with 20% fetal calf serum (FCS) (Sigma-Aldrich), 1% penicillin–streptomycin (Gibco) and fungizone (Gibco), and 0.1% ciprofloxacin (provided by apothecary LUMC) and gentamicin (Invitrogen), and immediately cut into small fragments in a Petri dish. Enzymatic digestion was performed using 1 mg ml^−1^ collagenase D (Roche Diagnostics) and 50 μg ml^−1^ DNase I (Roche Diagnostics) in 5 ml of IMDM + GlutaMax medium for 30 min at 37 °C in gentleMACS C tubes (Miltenyi Biotec). During and after incubation, cell suspensions were dissociated mechanically on the gentleMACS Dissociator (Miltenyi Biotec). Cell suspensions were filtered through a 70 μm cell strainer (Corning), washed in IMDM + GlutaMax medium with 20% FCS, 1% penicillin–streptomycin and 0.1% fungizone, and the cell count and viability were determined using the Muse Count & Viability Kit (Merck) on the Muse Cell Analyser (Merck). On the basis of the number of viable cells, cells in IMDM + GlutaMax medium were cryopreserved in liquid nitrogen until time of analysis complemented 1:1 with 80% FCS and 20% dimethyl sulfoxide (Merck).

### Immunohistochemical detection of MMR, B2M and HLA class I proteins

For the tumour tissue samples from the LUMC, tumour MMR status was determined by immunohistochemical detection of PMS2 (anti-PMS2 antibodies; EP51, DAKO) and MSH6 (anti-MSH6 antibodies; EPR3945, Abcam) proteins^53^. MMR-deficiency was defined as the lack of expression of at least one of the MMR-proteins in the presence of an internal positive control. Tumour B2M status was determined by immunohistochemical detection of B2M (anti-B2M antibodies; EP2978Y, Abcam). Immunohistochemical detection of HLA class I expression on tumour cells was performed with HCA2 and HC10 monoclonal antibodies (Nordic-MUbio), and tumours were classified as HLA class I positive, weak or loss, as described previously^16^. For the tumour samples from the NICHE study, immunohistochemistry analysis of the formalin-fixed paraffin-embedded (FFPE) tissue was performed on the BenchMark Ultra autostainer (Ventana Medical Systems). In brief, paraffin sections were cut at 3 μm, heated at 75 °C for 28 min and deparaffinized in the instrument using EZ prep solution (Ventana Medical Systems). Heat-induced antigen retrieval was performed using Cell Conditioning 1 (CC1, Ventana Medical Systems) for 32 min at 95 °C (HC10) or 64 min at 95 °C (B2M and HCA2). HLA class I heavy chain expression was detected using clone HCA2 (1:5,000, 60 min at room temperature; Nordic-Mubio) and clone HC10 (1:20,000, 32 min at 37 °C; Nordic-Mubio). B2M was detected using clone D8P1H (1:1,500, 60 min at room temperature; Cell Signaling). Bound antibodies were detected using the OptiView DAB Detection Kit (Ventana Medical Systems). Slides were counterstained with haematoxylin and Bluing Reagent (Ventana Medical Systems). A PANNORAMIC 1000 scanner from 3DHISTECH was used to scan the slides at ×40 magnification.

### Sorting of γδ T cells from colon cancers and scRNA-seq

scRNA-seq was performed on sorted γδ T cells from colon cancers (MMR-d) of five patients from the LUMC in the presence of hashtag oligos (HTOs) for sample ID and antibody-derived tags (ADTs) for CD45RA and CD45RO protein expression by CITE-seq^54^. Cells were thawed, rested at 37 °C in IMDM (Lonza)/20% FCS for 1 h, and then incubated with human Fc receptor block (BioLegend) for 10 min at 4 °C. Cells were then stained with cell surface antibodies (anti-CD3-PE (1:50, SK7, BD Biosciences), anti-CD45-PerCP-Cy5.5 (1:160, 2D1, eBioscience), anti-CD7-APC (1:200, 124-1D1, eBioscience), anti-EPCAM-FITC (1:60, HEA-125, Miltenyi), anti-TCRγδ-BV421 (1:80, 11F2, BD Biosciences); and a 1:1,000 near-infrared viability dye (Life Technologies)), 1 μg of TotalSeq-C anti-CD45RA (HI100, BioLegend, 2 μl per sample) and 1 μg of TotalSeq-C anti-CD45RO (UCHL1, BioLegend, 2 μl per sample) antibodies, and 0.5 μg of a unique TotalSeq-C CD298/B2M hashtag antibody (LNH-94/2M2, BioLegend, 1 μl per sample) for 30 min at 4 °C. Cells were washed three times in FACS buffer (PBS (Fresenius Kabi)/1% FCS) and kept under cold and dark conditions until cell sorting. Compensation was performed using CompBeads (BD Biosciences) and ArC reactive beads (Life Technologies). Single, live CD45^+^EPCAM^–^CD3^+^TCRγδ^+^ cells were sorted on the FACS Aria III 4L (BD Biosciences) system. After sorting, the samples were pooled.

scRNA-seq libraries were prepared using the Chromium Single Cell 5′ Reagent Kit v1 chemistry (10x Genomics) according to the manufacturer’s instructions. The construction of 5′ gene expression libraries enabled the identification of γδ T cell subsets according to Vδ and Vγ usage. Libraries were sequenced on the HiSeq X Ten using paired-end 2 × 150 bp sequencing (Illumina). Reads were aligned to the human reference genome (GRCh38) and quantified using Cell Ranger (v.3.1.0). Downstream analysis was performed using Seurat (v.3.1.5) according to the author’s instructions^55^. In brief, cells that had less than 200 detected genes and genes that were expressed in less than six cells were excluded. The resulting 5,669 cells were demultiplexed on the basis of HTO enrichment using the MULTIseqDemux algorithm^56^. Next, cells with a mitochondrial gene content of greater than 10% and cells with outlying numbers of expressed genes (>3,000) were filtered out from the analysis, resulting in a final dataset of 4,442 cells, derived from HTO1 (*n* = 332), HTO6 (*n* = 105), HTO7 (*n* = 1,100), HTO8 (*n* = 1,842) and HTO9 (*n* = 1,063). Data were normalized using the LogNormalize function of Seurat with a scale factor of 10,000. Variable features were identified using the FindVariableFeatures function of Seurat returning 2,000 features. We next applied the RunFastMNN function of SeuratWrappers split by sample ID to adjust for potential batch-derived effects across the samples^57^. Uniform manifold approximation and projection (UMAP)^58^ was used to visualize the cells in a two-dimensional space, followed by the FindNeighbors and FindClusters functions of Seurat. Data were scaled, and heterogeneity associated with mitochondrial contamination was regressed out. Cell clusters were identified by performing differentially expressed gene analysis using the FindAllMarkers function, with min.pct and logfc.threshold at 0.25. The number of *TRDV1*^*+*^ (Vδ1, *n* = 1,927), *TRDV2*^*+*^ (Vδ2, *n* = 860) or *TRDV3*^*+*^ (Vδ3, *n* = 506) cells was determined as the percentage of all cells with an expression level of >1, with <1 for the other TCR Vδ chains. CRC96, 134 and 167 had less than ten *TRDV3*^+^ cells, and were not included in the Vδ3 analysis. Transcripts of Vδ4 (*TRDV4*), Vδ5 (*TRDV5*) and Vδ8 (*TRDV8*) cells were not detected. The percentage of cells positive for a certain gene was determined as all cells with an expression level of >1.

### IMC staining and analysis

IMC analysis was performed on ICB-naive colon cancer tissues (MMR-d) of 17 patients from the LUMC; 5 of these colon cancer tissues had B2M defects and the remainder were B2M-positive (Supplementary Table 4). Moreover, IMC was performed on ICB-naive and ICB-treated colon cancer tissues (MMR-d) of ten patients from the NICHE study; five of these colon cancer tissues were *B2M*^*WT*^ and five were *B2M*^*MUT*^. Antibody conjugation and immunodetection were performed according to previously published methodology^59^. FFPE tissue (thickness, 4 μm) was incubated with 41 antibodies in four steps. First, sections were incubated overnight at room temperature with anti-CD4 and anti-TCRδ antibodies, which were subsequently detected using metal-conjugated secondary antibodies (1 μg ml^−1^, donkey anti-rabbit IgG and goat anti-mouse IgG, respectively; Abcam). Second, the sections were incubated with 20 antibodies (Supplementary Table 6) for five hours at room temperature. Third, the sections were incubated overnight at 4 °C with the remaining 19 antibodies (Supplementary Table 6). Fourth, the sections were incubated with 0.125 μM Cell-ID intercalator-Ir (Fluidigm) to detect the DNA, and stored dry until measurement. For each sample, six 1,000 μm × 1,000 μm regions (two to three for pretreatment NICHE biopsies due to the small tissue size) were selected on the basis of consecutive haematoxylin and eosin stains and ablated using the Hyperion Imaging system (Fluidigm). Data were acquired using the CyTOF Software (v.7.0) and exported using MCD Viewer (v.1.0.5). Data were normalized using semi-automated background removal in ilastik^60^ (v.1.3.3), to control for variations in the signal-to-noise ratio between FFPE sections as described previously^61^. Next, the phenotype data were normalized at the pixel level. Cell segmentation masks were created for all cells in ilastik and CellProfiler^62^ (v.2.2.0). In ImaCytE^63^ (v.1.1.4), cell segmentation masks and normalized images were combined to generate single-cell FCS files containing the relative frequency of positive pixels for each marker per cell. Cells forming visual neighbourhoods in a *t*-distributed stochastic neighbour embedding^64^ in Cytosplore^65^ (v.2.3.0) were grouped and exported as separate FCS files. The resulting subsets were imported back into ImaCyte and visualized on the segmentation masks. Expression of immunomodulatory markers was determined as all cells with a relative frequency of at least 0.2 positive pixels per cell. Differences in cells per mm^2^ were calculated using Mann–Whitney tests in GraphPad Prism (v.9.0.1). Image acquisition and analysis were performed blinded to group allocation.

### Sorting of γδ T cells from colon cancers and cell culturing

γδ T cells from colon cancers (MMR-d) of five patients from the LUMC were sorted for cell culture. Cells were thawed and rested at 37 °C in IMDM (Lonza)/10% nHS for 1 h. Next, cells were incubated with human Fc receptor block (BioLegend) and stained with cell surface antibodies (anti-CD3-Am Cyan (1:20, SK7, BD Biosciences), anti-TCRγδ-BV421 (1:80, 11F2, BD Biosciences) and anti-PD-1-PE (1:30, MIH4, eBioscience)) for 45 min at 4 °C together with different additional antibodies for immunophenotyping (anti-CD103-FITC (1:10, Ber-ACT8, BD Biosciences), anti-CD38-PE-Cy7 (1:200, HIT2, eBioscience); anti-CD39-APC (1:60, A1, BioLegend), anti-CD45RA-PE-Dazzle594 (1:20, HI100, Sony), anti-CD45RO-PerCP-Cy5.5 (1:20, UCHL1, Sony), anti-TCRαβ-PE-Cy7 (1:40, IP26, BioLegend), anti-TCRVδ1-FITC (1:50, TS8.2, Invitrogen) or anti-TCRVδ2-PerCP-Cy5.5 (1:200‘ B6, BioLegend). A 1:1,000 live/dead fixable near-infrared viability dye (Life Technologies) was included in each staining. Cells were washed three times in FACS buffer (PBS/1% FCS) and kept under cold and dark conditions until cell sorting. Compensation was performed using CompBeads (BD Biosciences) and ArC reactive beads (Life Technologies). Single, live CD3^+^TCRγδ^+^PD-1^+^ and PD-1^–^ cells were sorted on the FACS Aria III 4L (BD Biosciences) system. For CRC94, all γδ T cells were sorted owing to the low number of PD-1^+^ cells. γδ T cells were sorted in medium containing feeder cells (1 × 10^6^ per ml), PHA (1 μg ml^−1^; Thermo Fisher Scientific), IL-2 (1,000 IU ml^−1^; Novartis), IL-15 (10 ng ml^−1^; R&D Systems), gentamicin (50 μg ml^−1^) and fungizone (0.5 μg ml^−1^). Sorted γδ T cells were expanded in the presence of 1,000 IU ml^−1^ IL-2 and 10 ng ml^−1^ IL-15 for 3–4 weeks. The purity and phenotype of γδ T cells were assessed using flow cytometry. We obtained a >170,000-fold increase in 3–4 weeks of expansion of γδ T cells (Extended Data Fig. 4c).

### Immunophenotyping of expanded γδ T cells by flow cytometry

Expanded γδ T cells from colon tumours were analysed by flow cytometry for the expression of TCR Vδ chains, NKG2 receptors, NCRs, KIRs, tissue-residency/activation markers, cytotoxic molecules, immune checkpoint molecules, cytokine receptors and Fc receptors. In brief, cells were incubated with human Fc receptor block (BioLegend) and stained with cell surface antibodies (Supplementary Table 7) for 45 min at 4 °C, followed by three wash steps in FACS buffer (PBS/1% FCS). Granzyme B and perforin were detected intracellularly using fixation buffer and intracellular staining permeabilization wash buffer (BioLegend). Compensation was performed using CompBeads (BD Biosciences) and ArC reactive beads (Life Technologies). Cells were acquired on the FACS LSR Fortessa 4L (BD Biosciences) system running FACSDiva software (v.9.0; BD Biosciences). Data were analysed using FlowJo (v.10.6.1; Tree Star).

### Cancer cell line models and culture

Human colorectal adenocarcinoma cell lines HCT-15 (MMR-d), LoVo (MMR-d), HT-29 (MMR-p), SW403 (MMR-p) and SK-CO-1 (MMR-p), as well as HLA-class-I-deficient human leukaemia cell line K-562 and Burkitt lymphoma cell line Daudi were used as targets for reactivity and immune cell killing assays. All of the cell lines were obtained from the ATCC. The cell lines were authenticated by STR profiling and tested for mycoplasma. HCT-15, LoVo, HT-29, K-562 and Daudi cells were maintained in RPMI (Gibco)/10% FCS. SW403 and SK-CO-1 were maintained in DMEM/F12 (Gibco)/10% FCS. All adherent cell lines were trypsinized before passaging. The *B2M*-knockin HCT-15 and LoVo cell lines were generated using the *B2M* plasmid (pLV[Exp]-EF1A>hB2M[NM_004048.4](ns):T2A:Puro), produced in lentivirus according to standard methodology. Cells were selected using puromycin and then FACS-sorted on the basis of HLA-A/B/C expression using 1:100 anti-HLA-A/B/C-FITC (W6/32, eBioscience).

### Organoid models and culture

Tumour organoids were derived from MMR-d CRC tumours of two patients through resection from the colon (tumour organoid 1) or peritoneal biopsy (tumour organoid 2) (Supplementary Table 5). Establishment of the respective organoid lines from tumour material was performed as previously reported^66,67^. In brief, tumour tissue was mechanically dissociated and digested with 1.5 mg ml^−1^ of collagenase II (Sigma-Aldrich), 10 μg ml^−1^ of hyaluronidase type IV (Sigma-Aldrich) and 10 μM Y-27632 (Sigma-Aldrich). Cells were embedded in Cultrex RGF BME type 2 (3533-005-02, R&D systems) and placed into a 37 °C incubator for 20 min. Human CRC organoid medium is composed of Ad-DF+++ (Advanced DMEM/F12 (GIBCO) supplemented with 2 mM Ultraglutamine I (Lonza), 10 mM HEPES (GIBCO), 100 U ml^−1^ of each penicillin and streptomycin (GIBCO), 10% noggin-conditioned medium, 20% R-spondin1-conditioned medium, 1× B27 supplement without vitamin A (GIBCO), 1.25 mM *N*-acetylcysteine (Sigma-Aldrich), 10 mM nicotinamide (Sigma-Aldrich), 50 ng ml^−1^ human recombinant EGF (Peprotech), 500 nM A83-01 (Tocris), 3 μM SB202190 (Cayman Chemicals) and 10 nM prostaglandin E2 (Cayman Chemicals). Organoids were passaged depending on growth every 1–2 weeks by incubating in TrypLE Express (Gibco) for 5–10 min followed by embedding in BME. Organoids were authenticated by SNP array or STR analysis and were regularly tested for Mycoplasma using Mycoplasma PCR43 and the MycoAlert Mycoplasma Detection Kit (LT07-318). In the first two weeks of organoid culture, 1× Primocin (Invivogen) was added to prevent microbial contamination. Procedures performed with patient samples were approved by the Medical Ethical Committee of the Netherlands Cancer Institute–Antoni van Leeuwenhoek hospital (NL48824.031.14) and written informed consent was obtained from all of the patients. Mismatch repair status was assessed using a standard protocol for the Ventana automated immunostainer for MLH1 clone M1 (Roche), MSH2 clone G219-1129 (Roche), MSH6 clone EP49 (Abcam) and PMS2 clone EP51 (Agilant Technologies). The *B2M*^*KO*^ tumour organoid lines were generated using sgRNA targeting *B2M* (GGCCGAGATGTCTCGCTCCG), cloned into LentiCRISPR v2 plasmid. The virus was produced using a standard method.

### Screening of cancer cell lines and tumour organoids by flow cytometry

The cancer cell lines used in the reactivity and killing assays were screened for the expression of B2M, HLA class I molecules, NKG2D ligands, DNAM-1 ligands and butyrophilin using flow cytometry. In brief, cells were incubated with human Fc receptor block (BioLegend) and stained with the cell surface antibodies in different experiments (anti-CD112-PE (1:10, R2.525, BD Biosciences), anti-CD155-PE (1:10, 300907, R&D Systems), anti-CD277/BTN3A1-PE (1:50, BT3.1, Miltenyi), anti-B2M-PE (1:100, 2M2, BioLegend), anti-HLA-A/B/C-FITC (1:100, W6/32, eBioscience), anti-HLA-A/B/C-AF647 (1:160, W6/32, BioLegend), anti-HLA-E-BV421 (1:20, 3D12, BioLegend), anti-HLA-G-APC (1:20, 87G, BioLegend), anti-MICA/B-PE (1:300, 6D4, BioLegend), anti-ULBP1-PE (1:10, 170818, R&D Systems), anti-ULBP2/5/6-PE (1:20, 165903, R&D Systems), anti-ULBP3-PE (1:20, 166510, R&D Systems) or anti-ULBP4-PE (1:20, 709116, R&D Systems)) for 45 min at 4 °C. A 1:1,000 live/dead fixable near-infrared viability dye (Life Technologies) was included in each staining. Cells were washed three times in FACS buffer (PBS/1% FCS). Compensation was performed using CompBeads (BD Biosciences) and ArC reactive beads (Life Technologies). Cells were acquired on the FACS Canto II 3L or FACS LSR Fortessa 4L (BD Biosciences) system running FACSDiva software (v.9.0; BD Biosciences). Isotype or FMO controls were included to determine the percentage of positive cancer cells. Data were analysed using FlowJo v.10.6.1 (Tree Star).

For organoid surface staining, tumour organoids were dissociated into single cells using TrypLE Express (Gibco), washed twice in cold FACS buffer (PBS, 5 mM EDTA, 1% bovine serum antigen), and stained with anti-HLA-A/B/C-PE (1:20, W6/32, BD Biosciences), anti-B2M-FITC (1:100, 2M2, BioLegend), anti-PD-L1-APC (1:200, MIH1, eBioscience) and 1:2,000 near-infrared (NIR) viability dye (Life Technologies), or isotype controls (1:1,000 FITC; 1:20, PE; or 1:200, APC) mouse IgG1 kappa (BD Biosciences). For NKG2D ligand expression analysis, cells were stained with anti-MICA/MICB (1:300), anti-ULBP1 (1:10), anti-ULBP2/5/6 (1:20), anti-ULBP3 (1:20), anti-ULBP4 (1:20) and 1:2,000 near-infrared (NIR) viability dye (Life Technologies). Tumour cells were incubated for 30 min at 4 °C in the dark and washed twice with FACS buffer. All of the samples were recorded with the BD LSR Fortessa Cell Analyzer SORP flow cytometer using FACSDiVa (v.8.0.2; BD Biosciences). Data were analysed using FlowJo (v.10.6.1; BD) and presented using GraphPad Prism (v.9.0.0; GraphPad).

### Reactivity assay of γδ T cells

The reactivity of γδ T cells to the different cancer cell lines was assessed by a co-culture reactivity assay. γδ T cells were thawed and cultured in IMDM + GlutaMax (Gibco)/8% nHS medium with penicillin (100 IU ml^−1^) and streptomycin (100 μg ml^−1^) in the presence of low-dose IL-2 (25 IU ml^−1^) and IL-15 (5 ng ml^−1^) overnight at 37 °C. Cancer cell lines were counted, adjusted to a concentration of 0.5 × 10^5^ cells per ml in IMDM + GlutaMax/10% FCS medium with penicillin (100 IU ml^−1^) and streptomycin (100 μg ml^−1^), and seeded (100 μl per well) in coated 96-well flat-bottom microplates (Greiner CellStar) (for 5,000 cells per well) overnight at 37 °C. The next day, γδ T cells were collected, counted and adjusted to a concentration of 1.2 × 10^6^ cells per ml in IMDM + GlutaMax/10% FCS medium. The γδ T cells were added in 50 μl (for 60,000 cells per well) and co-cultured (12:1 effector:target ratio) at 37 °C for 18 h in biological triplicates. The medium (without cancer cells) was used as a negative control and PMA (20 ng ml^−1^)/ionomycin (1 μg ml^−1^) was used as a positive control. After co-culture, the supernatant was collected to detect IFNγ secretion by enzyme-linked immunosorbent assay (Mabtech) according to the manufacturer’s instructions. Moreover, cells were collected, incubated with human Fc receptor block (BioLegend) and stained with cell surface antibodies (anti-CD137-APC (1:100, 4B4-1, BD Biosciences), anti-CD226/DNAM-1-BV510 (1:150, DX11, BD Biosciences), anti-CD3-AF700 (1:400, UCHT1, BD Biosciences), anti-CD39-APC (1:80, A1, BioLegend), anti-CD40L-PE (1:10, TRAP1, BD Biosciences), or anti-PD-1-PE (1:30, MIH4, eBioscience), anti-TCRγδ-BV650 (1:40, 11F2, BD Biosciences), anti-NKG2D-PE-Cy7 (1:300, 1D11, BD Biosciences) and anti-OX40-FITC (1:20, ACT35, BioLegend)) for 45 min at 4 °C. A 1:1,000 live/dead fixable near-infrared viability dye (Life Technologies) was included in each staining. Cells were washed three times in FACS buffer (PBS/1% FCS). Compensation was performed using CompBeads (BD Biosciences) and ArC reactive beads (Life Technologies). Cells were acquired on the FACS LSR Fortessa X-20 4L (BD Biosciences) system running FACSDiva software (v.9.0; BD Biosciences). Data were analysed using FlowJo (v.10.6.1; Tree Star). All data are representative of at least two independent experiments.

### Immune cell killing assay γδ T cells

Killing of the different cancer cell lines by γδ T cells was visualized and quantified by a co-culture immune cell killing assay using the IncuCyte S3 Live-Cell Analysis System (Essen Bioscience). HCT-15, LoVo and HT-29 cells were transduced with IncuCyte NucLight Red Lentivirus Reagent (EF-1α, Puro; Essen BioScience) providing a nuclear-restricted expression of a red (mKate2) fluorescent protein. In brief, HCT-15, LoVo and HT-29 cells were seeded, transduced according to the manufacturer’s instructions and stable cell populations were generated using puromycin selection. The *B2M*-knockin cell lines were created under puromycin selection; therefore, stable NucLight Red-expressing cell populations were generated by sorting for mKate2 (the red fluorescent protein) in the PE Texas Red filter set instead. Cancer cell lines were counted, adjusted to a concentration of 1 × 10^5^ cells per ml in IMDM + GlutaMax/10% FCS medium with penicillin (100 IU ml^−1^) and streptomycin (100 μg ml^−1^), and seeded (100 μl per well) in 96-well flat-bottom clear microplates (Greiner CellStar) (for 10,000 cells per well). The target cell plate was placed in the IncuCyte system at 37 °C to monitor for cell confluency for 3 days. On day 2, γδ T cells were thawed and cultured in IMDM + GlutaMax/8% nHS medium with penicillin (100 IU ml^−1^) and streptomycin (100 μg ml^−1^) in the presence of low-dose IL-2 (25 IU ml^−1^) and IL-15 (5 ng ml^−1^) overnight at 37 °C. The next day, γδ T cells were collected, counted and adjusted to a concentration of 7.2 × 10^5^ cells per ml in IMDM + GlutaMax/10% FCS medium. After aspiration of the medium of the target cell plate, 100 μl of new medium containing 3.75 μM IncuCyte Caspase-3/7 Green Apoptosis Reagent (Essen BioScience) (1.5× final assay concentration of 2.5 μM) was added together with 50 μl of γδ T cells (for 36,000 cells per well). They were co-cultured (4:1 effector:target ratio) in the IncuCyte system at 37 °C in biological duplicates. Cancer cells alone and cancer cells alone with caspase-3/7 were used as negative controls. Images (2 images per well) were captured every hour at ×20 magnification with the phase, green and red channels for up to 4 days.

Analysis was performed using the IncuCyte software (v.2020B) for each cancer cell line separately. The following analysis definitions were applied: a minimum phase area of 200 μm^2^, RCU of 2.0, and a GCU of 2.0 (for HCT-15 cells) and 4.0 (for LoVo and HT-29 cells). Cancer cell apoptosis was then quantified in the IncuCyte software by counting the total number of green + red objects per image normalized (by division) to the total number of red objects per image after 12 h co-culture and displayed as a percentage (mean ± s.e.m.) of two wells with two images per well. For the comparison of the killing of *B2M*-knockin HCT-15 and LoVo cell lines versus the WT cell lines, Caspase-3/7 Red Apoptosis Reagent (Essen BioScience) was used. The transfection of the target reporter was not as successful in combination with the *B2M*-knockin. Thus, apoptosis was quantified by dividing the red area by the phase area and displayed as a percentage (mean ± s.e.m.) of two wells with two images per well. The following analysis definitions were applied: a minimum phase area of 100 μm^2^ and a RCU of 0.5 (for HCT-15 cells) and 0.75 (for LoVo cells).

### Tumour organoid recognition assay

For evaluation of tumour reactivity towards *B2M*^*WT*^ and *B2M*^*KO*^ organoids and NKG2D ligand blocking conditions, tumour organoids and γδ T cells were prepared as described previously^9,66,67^. Two days before the experiment, organoids were isolated from BME by incubation in 2 mg ml^−1^ type II dispase (Sigma-Aldrich) for 15 min before addition of 5 mM EDTA and washed with PBS before being resuspended in CRC organoid medium with 10 μM Y-27632 (Sigma-Aldrich). The organoids were stimulated with 200 ng ml^−1^ IFNγ (Peprotech) 24 h before the experiment. For the recognition assay and intracellular staining, tumour organoids were dissociated into single cells and plated in anti-CD28-coated (CD28.2, eBioscience) 96-well U-bottom plates with γδ T cells at a 1:1 target:effector ratio in the presence of 20 μg ml^−1^ anti-PD-1 (Merus). As a positive control, γδ T cells were stimulated with 50 ng ml^−1^ of phorbol-12-myristate-13-acetate (Sigma-Aldrich) and 1 μg ml^−1^ of ionomycin (Sigma-Aldrich). After 1 h of incubation at 37 °C, GolgiSTOP (BD Biosciences, 1:1,500) and GolgiPlug (BD Biosciences, 1:1,000) were added. After 4 h of incubation at 37 °C, γδ T cells were washed twice in cold FACS buffer (PBS, 5 mM EDTA, 1% bovine serum antigen) and stained with anti-CD3-PerCP-Cy5.5 (1:20, BD Biosciences), anti-TCRγδ-PE (1:20, BD Bioscience), anti-CD4-FITC (1:20, BD Bioscience) (not added in experiments with NKG2D ligand blocking), anti-CD8-BV421 (1:200, BD Biosciences) and 1:2,000 near-infrared (NIR) viability dye (Life Technologies) for 30 min at 4 °C. Cells were washed, fixed and stained with 1:40 anti-IFNγ-APC (BD Biosciences) for 30 min at 4 °C, using the Cytofix/Cytoperm Kit (BD Biosciences). After two wash steps, cells were resuspended in FACS buffer and recorded with the BD LSR Fortessa Cell Analyzer SORP flow cytometer using FACSDiVa software (v.8.0.2; BD Biosciences). Data were analysed using FlowJo (v.10.6.1, BD) and presented using GraphPad Prism (v.9.0.0, GraphPad).

### Blocking experiments with cancer cell lines and tumour organoids

The reactivity of and killing by the γδ T cells was examined in the presence of different blocking antibodies to investigate which receptor–ligand interactions were involved. For DNAM-1 blocking, γδ T cells were incubated with 3 μg ml^−1^ purified anti-DNAM-1 (DX11, BD Biosciences) for 1 h at 37 °C. For γδ TCR blocking, γδ T cells were incubated with 3 μg ml^−1^ purified anti-TCRγδ (5A6.E9, Invitrogen) for 1 h at 37 °C; the clone that we used was tested to be the best for use in γδ TCR blocking assays^68^. NKG2D ligands were blocked on the cancer cell lines and single cells of tumour organoids by incubating the target cells with 12 μg ml^−1^ anti-MICA/B (6D4, BioLegend), 1 μg ml^−1^ anti-ULBP1 (170818, R&D Systems), 3 μg ml^−1^ anti-ULBP2/5/6 (165903, R&D Systems) and 6 μg ml^−1^ anti-ULBP3 (166510, R&D Systems) for 1 h at 37 °C before plating with γδ T cells. After incubation with the blocking antibodies, the γδ T cells were added to cancer cell lines HCT-15, LoVo and HT-29 as described above with a minimum of two biological replicates per blocking condition. For organoid experiments, anti-CD107a-FITC (1:50, H4A3, BioLegend) was added during incubation.

As a control for Fc-mediated antibody effector functions, γδ T cells alone were incubated with the blocking antibodies in the presence of 2.5 μM IncuCyte Caspase-3/7 Green Apoptosis Reagent (Essen BioScience) in the IncuCyte system at 37 °C, and the number of apoptotic γδ T cells was quantified over time.

### Data analysis and visualization

Bulk DNA-seq and RNA-seq data were analysed using Python (v.3) and R (v.3.6.1) in Jupyter Notebook (v.6.0.1). Numpy (v.1.17.2) and Pandas (v.0.25.1) were used for array and data frame operations, respectively. Data visualization was performed using Matplotlib (v.3.2.1) and Seaborn (v.0.9.0). scRNA-seq data were analysed using Cell Ranger (v.3.1.0), R (v.4.1.0) and Seurat (v.3.1.5). IMC data were analysed using ilastik (v.1.3.3), CellProfiler (v.2.2.0), ImaCytE (v.1.1.4) and Cytosplore (v.2.3.0). Flow cytometry data were analysed using FlowJo (v.10.6.1). IncuCyte data were analysed using IncuCyte (v.2020B). Data visualization was performed using GraphPad Prism (v.9.0.0 and v.9.0.1).

### Reporting summary

Further information on research design is available in the Nature Portfolio Reporting Summary linked to this article.

## Online content

Any methods, additional references, Nature Portfolio reporting summaries, source data, extended data, supplementary information, acknowledgements, peer review information; details of author contributions and competing interests; and statements of data and code availability are available at 10.1038/s41586-022-05593-1.

## Supplementary information

Supplementary Information A guide to the Supplementary Information, and Supplementary Tables 4–7.

Reporting Summary

Supplementary Table 1 Characteristics of clinical samples from 71 patients with MMR-d cancers from the DRUP study.

Supplementary Table 2 Immune marker gene sets for bulk RNA-seq analyses.

Supplementary Table 3 Characteristics of clinical samples from 2,256 patients with MMR-p cancers from the Hartwig database.

Supplementary Video 1 Killing of CRC cells after co-culture with γδ T cells. Killing of HCT-15 cells (red) by γδ T cells (Vδ1^+^) from a MMR-d colon cancer in the presence of caspase-3/7 (green). γδ T cells were added at *T* = 6 h, and images were taken until 12 h after (*T* = 18 h). Cancer cell apoptosis is visualized in yellow.

Supplementary Video 2 Pronounced cancer cell apoptosis after co-culture with PD-1^+^ (Vδ1^+^) as compared to PD-1^–^ γδ T cells. Killing of HCT-15 cells (red) by PD-1^+^ (Vδ1^+^, left) as compared to PD-1^–^ (Vδ2^+^, right) γδ T cells from a MMR-d colon cancer in the presence of caspase-3/7 (green). γδ T cells were added at *T* = 5 h, and images were taken until 12 h after (*T* = 17 h). Cancer cell apoptosis is visualized in yellow.

Peer Review File

## Data Availability

The TCGA data used here are publicly available at the National Cancer Institute GDC Data Portal (https://portal.gdc.cancer.gov; cohorts COAD, STAD and UCEC). Of the DRUP study participants included in this preliminary analysis across all (complete and incomplete) cohorts of the study, we included all clinical data, genomics data on *B2M* status and RNA expression data of marker gene sets in Supplementary Table 1. The raw sequencing data of the DRUP and Hartwig cohorts can be accessed through Hartwig Medical Foundation on approval of a research access request (https://www.hartwigmedicalfoundation.nl/en/data/data-acces-request). As determined in the original publication, NICHE study RNA-seq and DNA-seq data have been deposited into the European Genome–Phenome Archive under accession number EGAS00001004160 and are available on reasonable request for academic use and within the limitations of the provided informed consent. The scRNA-seq data have been deposited at the GEO (GSE216534) and are publicly available. All other data are available from the corresponding author on reasonable request. The GRCh38 primary assembly of the human reference genome was downloaded from Gencode (https://ftp.ebi.ac.uk/pub/databases/gencode/Gencode_human/release_42/GRCh38.primary_assembly.genome.fa.gz) with Gencode’s matching v29 annotation file (https://ftp.ebi.ac.uk/pub/databases/gencode/Gencode_human/release_29/gencode.v29.annotation.gtf.gz) for gene expression quantification.
