# Supplementary material for: γδ T cells are effectors of immunotherapy in cancers with HLA class I defects
Source: Nature. 2023 Jan 11;613(7945):743–50. doi: 10.1038/s41586-022-05593-1 (PMC9876799; doi:10.1038/s41586-022-05593-1)
Supplement: Supplementary file 1 — A guide to the Supplementary Information, and Supplementary Tables 4–7. [file 41586_2022_5593_MOESM1_ESM.pdf]

nature portfolio

https://doi.org/10.1038/s41586-022-05593-1

Supplementary information

γδ T cells are effectors of immunotherapy in cancers with HLA class I defects

In the format provided by the authors and unedited

Supplementary information

Supplemental Table 1. Characteristics of clinical samples from 71 patients with MMR-d cancers from the DRUP study.

See separate - Excel table

Supplemental Table 2. Immune marker gene sets for bulk RNA-seq analyses.

See separate - Excel table

Supplemental Table 3. Characteristics of clinical samples from 2,256 patients with MMR-p cancers from the Hartwig database.

See separate - Excel table

Supplemental Table 4. Characteristics of clinical samples from 17 patients with ICB-naïve MMR-d colon cancer.

Patient ID	Tumor location	HLA class I status	β2m status	*B2M* status	Subgroup
CRC 1	Flexura lienalis	Positive	Positive	Wildtype	$\beta 2m^{+}$
CRC 2	Coecum	Defect	Mut pattern	Wildtype	$\beta 2m^{+}$
CRC 3	Flexura hepatica	Positive	Positive	Wildtype	$\beta 2m^{+}$
CRC 6	Descendens	Defect	Defect	Mutated	$\beta 2m^{-}$
CRC 19	Ascendens	Defect	Defect	Mutated	$\beta 2m^{-}$
CRC 52	Ascendens	Defect	Mut pattern	Wildtype	$\beta 2m^{+}$
CRC 67	Transversum	Positive	Positive	Wildtype	$\beta 2m^{+}$
CRC 84	Sigmoid	Defect	Defect	Wildtype	$\beta 2m^{-}$
CRC 94 #	Sigmoid	Defect	Positive	Wildtype	$\beta 2m^{+}$
CRC 96* #	Transversum	Defect	Defect	Mutated	$\beta 2m^{-}$
CRC 102	Ascendens	Defect	Positive	Wildtype	$\beta 2m^{+}$
CRC 134* #	Ascendens	Defect	Defect	Mutated	$\beta 2m^{-}$
CRC 154* #	Flexura hepatica	Positive	Positive	Wildtype	$\beta 2m^{+}$
CRC 159*	Coecum	Defect	Mut pattern	Wildtype	$\beta 2m^{+}$
CRC 167* #	Ascendens	Defect	Mut pattern	Wildtype	$\beta 2m^{+}$
CRC 177	Flexura hepatica	Defect	Positive	Wildtype	$\beta 2m^{+}$
CRC 195	Flexura hepatica	Defect	Positive	Wildtype	$\beta 2m^{+}$

* Used for single-cell RNA-sequencing experiments, # Used for cell culturing experiments. *B2M*, β2-microglobulin; MMR, mismatch repair.

Supplemental Table 5. Characteristics of patient-derived organoids from MMR-d colorectal cancer.

**Sample**	**PDTO-1 WT**	**PDTO-1 *B2M* ko**	**PDTO-2 WT**	**PDTO-2 *B2M* ko**
**Tumor**	CRC	CRC	CRC	CRC
**MMR status**	deficient	deficient	deficient	deficient
**Biopsy / Resection**	Resection	Resection	Biopsy	Biopsy
**Biopsied lesion**	Colon	Colon	Peritoneal	Peritoneal
**Primary / Metastasis**	Primary	Primary	Metastasis	Metastasis
**Authenticated**	Confirmed by SNP	Confirmed by STR	Confirmed by SNP	Confirmed by SNP
**Mycoplasma**	Negative	Negative	Negative	Negative
**Characteristics**	Luciferase transduced; parental line of PDTO-1 *B2M*-KO	*B2M*-knockout of PDTO-1 WT	Parental line of PDTO-2 *B2M*-KO	*B2M*-knockout of PDTO-2 WT
***B2M* knockout**	-	sgRNA targeting *B2M* (GGCCGAGATGTCTCGCTCCG) cloned into LentiCRISPR v2 plasmid	-	sgRNA targeting *B2M* (GGCCGAGATGTCTCGCTCCG) cloned into LentiCRISPR v2 plasmid

Supplemental Table 6. Antibodies used for imaging mass cytometry of colon cancers.

Antibody	Metal	Clone	Supplier	Catalog number	Lot number	Incubation time (temperature)	Dilution
β-catenin	89Y	D10A8	CST	8480BF	8	Overnight (4°C)	1:100
CD103	168 Er	EPR4166(2)	Abcam	ab221210	GR3355784-7	5h (RT)	1:50
CD11b	144 Nd	D6X1N	CST	49420BF	4	5h (RT)	1:100
CD11c	176 Yb	EP1347Y	Abcam	ab216655	GR3357092-9	5h (RT)	1:100
CD14	163 Dy	D7A2T	CST	56082BF	2	5h (RT)	1:100
CD15	171 Yb	MC480	CST	4744BF	5	Overnight (4°C)	1:100
CD163	173 Yb	EPR14643-36	Abcam	93498BF	Not available	5h (RT)	1:50
CD20	142 Nd	E7B7T	CST	48750BF	9179056	Overnight (4°C)	1:100
CD204	164 Dy	J5HTR3	Thermo Fisher Scientific	14-9054-95	4338161	5h (RT)	1:50
CD3	153 Eu	EP449E	Abcam	ab271850	GR3341846-3	Overnight (4°C)	1:50
CD31	147 Sm	89C2	CST	3528BF	Not available	Overnight (4°C)	1:100
CD38	169 Tm	EPR4106	Abcam	ab226034	GR3378690-1	Overnight (4°C)	1:100
CD39	157 Gd	EPR20627	Abcam	ab236038	GR3274485-6	5h (RT)	1:100
CD4*	145 Nd	EPR6855	Abcam	ab181724	GR3285644-10	Overnight (RT)	1:100
CD45	149 Sm	D9M8I	CST	13917BF	11	Overnight (4°C)	1:50
CD45RO	165 Ho	UCHL1	CST	55618BF	2	Overnight (4°C)	1:100
CD56	167 Er	E7X9M	CST	99746BF	2	5h (RT)	1:100
CD57	151 Eu	HNK-1 / Leu-7	Abcam	ab269781	GR3373313-3	Overnight (4°C)	1:100
CD68	143 Nd	D4B9C	CST	76437BF	2	Overnight (4°C)	1:100
CD7	174 Yb	EPR4242	Abcam	ab230834	Not available	5h (RT)	1:100
CD8α	146 Nd	D8A8Y	CST	85336BF	Not available	5h (RT)	1:50
Cleaved caspase-3	172 Yb	5A1E	CST	9664BF	24	5h (RT)	1:100
D2-40	166 Er	D2-40	BioLegend	916606	B316467	Overnight (4°C)	1:100
FOXP3	159 Tb	D608R	CST	12653BF	8	Overnight (4°C)	1:50
Granzyme B	150 Nd	D6E9W	CST	46890BF	3	5h (RT)	1:100
Histone H3	209	D1H2	CST	4499BF	Not available	Overnight (4°C)	1:50
HLA-DR	141 Pr	TAL 1B5	Abcam	ab176408	GR3384096-1	5h (RT)	1:100
ICOS	161 Dy	D1K2T™	CST	89601BF	4	5h (RT)	1:50
IDO	162 Dy	D5J4E™	CST	86630BF	7	Overnight (4°C)	1:100
Ki-67	152 Sm	8D5	CST	9449BF	11	Overnight (4°C)	1:100

LAG-3	155 Gd	D2G40™	CST	15372BF	Not available	5h (RT)	1:50
p16ink4a	175 Lu	D3W8G	CST	92803BF	2	Overnight (4°C)	1:100
Pan-keratin	198 Pt	C11 and AE1/AE3	CST / BioLegend	4545BF / 914204	12 / B302316	Overnight (4°C)	1:50
PD-1	160 Gd	D4W2J	CST	86163BF	7	5h (RT)	1:50
PD-L1	156 Gd	E1L3N®	CST	13684BF	17	Overnight (4°C)	1:50
T-bet	170 Er	4B10	BioLegend	644825	B298378	5h (RT)	1:50
TCRδ*	148 Nd	H41	Santa Cruz	sc-100289	D3021	Overnight (RT)	1:50
TGFβ	115 In	TB21	Thermo Fisher Scientific	MA5-16949	151471	5h (RT)	1:100
TIM-3	154 Sm	D5D5R™	CST	45208BF	9	5h (RT)	1:100
Vimentin	194 Pt	D21H3	CST	5741BF	9	Overnight (4°C)	1:50
VISTA	158 Gd	D1L2G™	CST	64953BF	7	5h (RT)	1:100

* Detection with metal-conjugated secondary antibodies. CST, Cell Signaling Technology.

Supplemental Table 7. Antibodies used for immunophenotyping of $\gamma\delta$ T cells by flow cytometry.

Antibody	Fluorochrome	Clone	Supplier	Catalog number	Lot number	Dilution
CD16	PE	B73.1	BD Biosciences	332779	9045985	1:60
CD103	FITC	Ber-ACT8	BD Biosciences	550259	2332847	1:10
CD122/IL-2Rβ	BV421	TU27	BioLegend	339010	B313155	1:20
CD132/IL-2Rγ	APC	TUGh4	BioLegend	338608	B293032	1:80
CD161	BV605	DX12	BD Biosciences	563863	7030586	1:20
CD25/IL-2Rα	PE-Cy7	M-A251	BD Biosciences	557741	9301660	1:25
CD215/IL-15Rα	PE	JM7A4	BioLegend	330208	B265801	1:80
CD226/DNAM-1	BV510	DX11	BD Biosciences	742494	9203072	1:150
CD3	Am Cyan	SK7	BD Biosciences	339186	9161745	1:20
CD32	APC	FLI8.26	BD Biosciences	559769	184743	1:20
CD38	PE-Cy7	HIT2	eBioscience	25-0389-42	4319912	1:200
CD39	APC	A1	BioLegend	328210	B249211	1:60
CD45RA	FITC	L48	BD Biosciences	335039	8227525	1:30
CD45RA	PE-Dazzle594	HI100	Sony	2120730	126470	1:20
CD45RO	PerCP-Cy5.5	UCHL1	Sony	2121110	138351	1:20
CD56	APC-R700	NCAM16.2	BD Biosciences	565139	5251693	1:150
CD64	FITC	10.1	BD Biosciences	555527	58058	1:20
CD69	PerCP-Cy5.5	FN50	BioLegend	310925	B266970	1:200
CD8α	BV605	SK1	BD Biosciences	564115	7092	1:100
CD94	BV605	HP-3D9	BD Biosciences	743950	7138571	1:200
GITR	PE	108-17	BioLegend	371204	B244963	1:50
Granzyme B*	PE	GB11	eBiosciences	12-8899-41	1928380	1:50
KIR2DL1	PE	HP3-E4	BD Biosciences	556063	86798	1:20
KIR2DL1/S1	PE	EB6	Beckman Coulter	A09778	12	1:50
KIR2DL2/L3/S2	PE	GL183	Beckman Coulter	IM2278U	200051	1:50
KIR2DL4	PE	181703	R&D Systems	FAB2238P	AAHO0209081	1:10
KIR2DS4	PE	FES172	Beckman Coulter	IM3337	200037	1:40
KIR3DL1	PE	DX9	BD Biosciences	555967	121769	1:80
KIR3DL1/S1	PE	Z27	Beckman Coulter	IM3292	200044	1:20
KIR3DL2	PE	#539304	R&D Systems	FAB2878P	ADBO0217051	1:10
LAG3	PE-Cy7	11C3C65	BioLegend	369309	B289009	1:100
NKG2A	APC	z199	Beckman Coulter	A60797	200046	1:30
NKG2C	PE	134591	Beckman Coulter	FAB138P	LCN0818011	1:20
NKG2D	PE-Cy7	1D11	BD Biosciences	562365	9045733	1:300

NKp44	APC	P44-8	BioLegend	325109	B160899	1:20
NKp46	PE	9E2	BioLegend	331907	B150121	1:20
PD-1	PE	MIH4	eBioscience	12-9969-42	1952441	1:30
Perforin*	PE-Cy7	dG9	BioLegend	308125	B215704	1:20
TCRγδ	BV421	11F2	BD Biosciences	744870	9340519	1:80
TCRγδ	BV650	11F2	BD Biosciences	745359	7222894	1:40
TCR Vδ1	FITC	TS8.2	Invitrogen	TCR2730	UH286015	1:50
TCR Vδ2	PerCP-Cy5.5	B6	BioLegend	331424	B279957	1:200
TIGIT	APC	1D11	BD Biosciences	562365	9045733	1:300
Live/dead	nIR	n.a.	Life Technologies	L10119	1808830	1:1000

* Detected intracellularly

Videos (supplied separately):

Video 1. Killing of CRC cells upon co-culture with γδ T cells. Killing of HCT-15 cells (red) by γδ T cells (Vδ1$^+$) from a MMR-d colon cancer in the presence of caspase-3/7 (green). γδ T cells were added at T=6h, and images were taken until 12 hours after (T=18h). Cancer cell apoptosis is visualized in yellow.

Video 2. Pronounced cancer cell apoptosis upon co-culture with PD-1$^+$ (Vδ1$^+$) as compared to PD-1$^-$ γδ T cells. Killing of HCT-15 cells (red) by PD-1$^+$ (Vδ1$^+$, left panel) as compared to PD-1$^-$ (Vδ2$^+$, right panel) γδ T cells from a MMR-d colon cancer in the presence of caspase-3/7 (green). γδ T cells were added at T=5h, and images were taken until 12 hours after (T=17h). Cancer cell apoptosis is visualized in yellow.
