# Supplementary material for: γδ T cells are effectors of immunotherapy in cancers with HLA class I defects
Source: Nature. 2023 Jan 11;613(7945):743–50. doi: 10.1038/s41586-022-05593-1 (PMC9876799; doi:10.1038/s41586-022-05593-1)
Supplement: Supplementary file 2 — Reporting Summary [file 41586_2022_5593_MOESM2_ESM.pdf]

nature portfolio

Corresponding author(s): E. E. Voest, N.F.C.C. de Miranda

Last updated by author(s): Nov 8, 2022

Reporting Summary

Nature Portfolio wishes to improve the reproducibility of the work that we publish. This form provides structure for consistency and transparency in reporting. For further information on Nature Portfolio policies, see our Editorial Policies and the Editorial Policy Checklist.

Statistics

For all statistical analyses, confirm that the following items are present in the figure legend, table legend, main text, or Methods section.

n/a	Confirmed	
☐	☒	The exact sample size (*n*) for each experimental group/condition, given as a discrete number and unit of measurement
☐	☒	A statement on whether measurements were taken from distinct samples or whether the same sample was measured repeatedly
☐	☒	The statistical test(s) used AND whether they are one- or two-sided<br>*Only common tests should be described solely by name; describe more complex techniques in the Methods section.*
☐	☒	A description of all covariates tested
☐	☒	A description of any assumptions or corrections, such as tests of normality and adjustment for multiple comparisons
☐	☒	A full description of the statistical parameters including central tendency (e.g. means) or other basic estimates (e.g. regression coefficient) AND variation (e.g. standard deviation) or associated estimates of uncertainty (e.g. confidence intervals)
☐	☒	For null hypothesis testing, the test statistic (e.g. *F*, *t*, *r*) with confidence intervals, effect sizes, degrees of freedom and *P* value noted<br>*Give P values as exact values whenever suitable.*
☒	☐	For Bayesian analysis, information on the choice of priors and Markov chain Monte Carlo settings
☒	☐	For hierarchical and complex designs, identification of the appropriate level for tests and full reporting of outcomes
☒	☐	Estimates of effect sizes (e.g. Cohen's *d*, Pearson's *r*), indicating how they were calculated

Our web collection on statistics for biologists contains articles on many of the points above.

Software and code

Policy information about availability of computer code

Data collection	All data of The Cancer Genome Atlas (TCGA) Research Network were downloaded via the GDC data portal (https://portal.gdc.cancer.gov) on April 10th, 2019. DRUP study and Hartwig database mutation and copy number calls were generated as previously described (Priestley et al., 2019) with an optimized pipeline based on open source tools, which is freely available on GitHub (https://github.com/hartwigmedical/pipeline5). This pipeline also outputs genome-wide number of indels in microsatellites as determined by MSIseq version 1.0.0 (Huang et al, 2015), as an integrated functionality. These data (mutations, copy numbers, indels in microsatellites) and raw RNA reads (as FASTA files) were downloaded from Hartwig Medical Foundation via Google Cloud software. For These RNA reads were aligned to the human reference genome (GRCh38 primary assembly as distributed by Gencode) with STAR software (Dobin et al., 2013), version 2.7.7a, using default settings in two-pass mode and Gencode's v29 annotation file. Flow cytometry data were collected in BD FACSDiva software (versions 8.0.2 and 9.0). Single-cell RNA libraries were sequenced on a HiSeq X Ten using paired-end 2x150 bp sequencing (Illumina). Immune cell killing assays were performed in the IncuCyte S3 system and analyzed with IncuCyte Analysis Software (version 2020B). Imaging mass cytometry data were collected using CyTOF software (version 7.0), and exported with MCD Viewer (version 1.0.5). For NICHE study (ClinicalTrials.gov: NCT03026140) RNA sequencing data, we used raw RNA reads as FASTA files, which were generated as described in the original publication (Chalabi et al., 2020). For NICHE study somatic mutation data, we used DNA sequencing of pre-treatment tumor biopsies and matched germline DNA, as described in the original publication (Chalabi et al., 2020).
Data analysis	Python 3, Jupyter Notebook 6.0.1, STAR 2.7.7a, Numpy 1.17.2, Pandas 0.25.1, Scipy 1.3.1, Statsmodels 0.10.1, Matplotlib 3.2.1, Seaborn 0.9.0, R 3.6.1, EdgeR 3.28.1, Limma (including Voom) 3.42.2, Lme4 1.1.26, MSIseq version 1.0.0, FlowJo 10.6.1, Cell Ranger 3.1.0, R version 4.1.0, Seurat R package 3.1.5, IncuCyte software 2020B, ilastik 1.3.3, CellProfiler 2.2.0, ImaCytE 1.1.4, Cytosplore 2.3.0, GraphPad Prism 9.0.0 and 9.0.1.

For manuscripts utilizing custom algorithms or software that are central to the research but not yet described in published literature, software must be made available to editors and reviewers. We strongly encourage code deposition in a community repository (e.g. GitHub). See the Nature Portfolio guidelines for submitting code & software for further information.

Data

Policy information about availability of data

All manuscripts must include a data availability statement. This statement should provide the following information, where applicable:

- Accession codes, unique identifiers, or web links for publicly available datasets
- A description of any restrictions on data availability
- For clinical datasets or third party data, please ensure that the statement adheres to our policy

RNA expression data (raw counts) of the colon adenocarcinoma (COAD), stomach adenocarcinoma (STAD) and Uterus Corpus Endometrium Carcinoma (UCEC) cohorts of The Cancer Genome Atlas (TCGA) Research Network is publicly available via the National Cancer Institute GDC Data Portal (https://portal.gdc.cancer.gov; cohorts COAD [https://portal.gdc.cancer.gov/projects/TCGA-COAD], STAD [https://portal.gdc.cancer.gov/projects/TCGA-STAD] and UCEC [https://portal.gdc.cancer.gov/projects/TCGA-UCEC]). Of these cohorts, mutation, copy number, purity and ploidy data were downloaded from GDC on November 11th, 2021, as the controlled access ABSOLUTE-annotated MAF file (mutations), SNP6 whitelisted copy number segments file (copy numbers), and ABSOLUTE purity/ploidy file of the TCGA PanCanAtlas project (Taylor et al, 2018). Mismatch repair-deficiency status was obtained from Thorsson et al., 2019 (TCGA Subtype = GI.HM-indel or UCEC.MSI).

Of DRUP study subjects included in this preliminary analysis across all (complete and incomplete) cohorts of the study, we included all clinical data, genomics data on B2M status and RNA-expression data of marker gene sets to this manuscript in Supplemental Table 1. The raw sequencing data of the DRUP and Hartwig cohorts can be accessed through Hartwig Medical Foundation upon approval of a research access request (https://www.hartwigmedicalfoundation.nl/en/data/data-acces-request). As determined in the original publication, NICHE study RNA- and DNA-sequencing data is deposited into the European Genome–Phenome Archive under accession no. EGAS00001004160 and are available on reasonable request to the NICHE study team for academic use and within the limitations of the provided informed consent. The single-cell RNA-sequencing data is deposited into the GEO database under accession no. GSE216534 and is publicly available. The GRCh38 primary assembly of the human reference genome was downloaded from Gencode (https://ftp.ebi.ac.uk/pub/databases/gencode/Gencode_human/release_42/GRCh38.primary_assembly.genome.fa.gz) with Gencode's matching v29 annotation file (https://ftp.ebi.ac.uk/pub/databases/gencode/Gencode_human/release_29/gencode.v29.annotation.gtf.gz) for gene expression analysis.

Field-specific reporting

Please select the one below that is the best fit for your research. If you are not sure, read the appropriate sections before making your selection.

☒ Life sciences ☐ Behavioural & social sciences ☐ Ecological, evolutionary & environmental sciences

For a reference copy of the document with all sections, see nature.com/documents/nr-reporting-summary-flat.pdf

Life sciences study design

All studies must disclose on these points even when the disclosure is negative.

Sample size

In all analyses, sample sizes were determined by availability of patients and data. Hence, no statistical methods were used to predetermine sample sizes, but we included as many patients as possible according to availability for the different analyses.

Sample sizes were as follows:
TCGA colon adenocarcinoma (COAD; n=50 β2-microglobulin wild type (B2M-WT), n=7 β2-microglobulin mutant (B2M-MUT), stomach adenocarcinoma (STAD; n=48 B2M-WT, n=12 B2M-MUT), and endometrium carcinoma (UCEC; n=118 B2M-WT, n=4 B2M-MUT). LUMC imaging mass cytometry (IMC) cohort: n=17 treatment-naïve patients with mismatch repair-deficient (MMR-d) colon cancer. DRUP study: n=50 B2M-WT, n=21 B2M-MUT. Single-cell RNA-sequencing: n=5 MMR-d colon cancer patients. γδ T cell subsets culture: n=5 MMR-d colon cancer patients. NICHE IMC cohort: n=10 patients with MMR-d colon cancer (n=5 B2M-WT, n=5 B2M-MUT) from which samples were obtained before and after immune checkpoint blockade (ICB).

These sample sizes were based on previous experiences (Chalabi et al., Nat Med., 2020), and are in line with standards in the field (Middha et al., JCO Precis Oncol., 2019; Wu et al., Sci Transl Med., 2019; Wu et al., Nat Cancer., 2022).

Data exclusions

For the Hartwig dataset, we excluded 89 tumors from rare primary tumor locations, defined as locations with less that <20 patients in our selection. When individual patients had data available of biopsies obtained at different timepoints, we only included data of the first biopsy. In bulk transcriptomic analyses, the gene set “NK CD56dim cells” of Danaher et al. (comprising IL21R, KIR2DL3, KIR3DL1, and KIR3DL2) was excluded, as three out of four genes within this set were killer-cell immunoglobulin-like receptors (KIRs) and hence this set showed high collinearity/redundancy to the full KIR gene set. As XLC1 and XLC2 are highly expressed by tumor-infiltrating γδ T cells, these genes were removed from the NK cell marker gene set and replaced by KLRF1, which encodes the well-established NK cell marker NKp80. The resulting gene set consisted of NCR1 and KLRF1, encoding the well-established NK cell markers NKp46 and NKp80, respectively. Finally, we reduced the “cytotoxic cells” marker gene set of Danaher et al. to those genes in the set encoding cytotoxic molecules (GZMA, GZMB, GZMH, PRF1, GNLY, CTSW). For single-cell RNA-sequencing data, cells that had less than 200 detected genes and genes that were expressed in less than six cells were excluded.

Replication

All findings reported are replicable and the source of materials and experimental procedure are documented in detail in the Methods section. The in vitro reactivity data reported were reproducible and were shown for >= 2 independent experiments where possible (depending on availability of γδ T cells). Regarding reproducibility of the IMC data, six independent 1x1 mm Regions of Interest (ROIs) were analyzed for each tissue sample with the exception of the pre-treatment NICHE biopsies, where to to three ROIs were analyzed due to the small tissue size. For scRNA-seq data, γδ T cell clusters were identified in multiple patient samples.

Randomization	No randomization was applicable as this study was purely observational.
Blinding	In the DRUP study, response assessment was performed in a standardized manner by clinicians blinded to B2M status.<br>The TCGA-based differential expression analysis were standardized and unbiased (performed across all human genes) and hence did not require blinding.<br>The transcriptomics analyses of Hartwig, DRUP, and NICHE followed the same methodology as the TCGA-based analysis and were independent from judgement of individual researchers and hence did not require blinding.<br>IMC staining and analysis of the LUMC and NICHE (ICB-naive and ICB-treated) cohorts were performed blinded to group allocation.

Reporting for specific materials, systems and methods

We require information from authors about some types of materials, experimental systems and methods used in many studies. Here, indicate whether each material, system or method listed is relevant to your study. If you are not sure if a list item applies to your research, read the appropriate section before selecting a response.

Materials & experimental systems

n/a	Involved in the study
☐	☒ Antibodies
☐	☒ Eukaryotic cell lines
☒	☐ Palaeontology and archaeology
☒	☐ Animals and other organisms
☐	☒ Human research participants
☐	☒ Clinical data
☒	☐ Dual use research of concern

Methods

n/a	Involved in the study
☒	☐ ChIP-seq
☐	☒ Flow cytometry
☒	☐ MRI-based neuroimaging

Antibodies

Antibodies used

Immunohistochemistry tumor tissue samples LUMC:
Antibody, Clone, Supplier, Catalog number, Lot number, Dilution
PMS2, EP51, DAKO, M364729-2, 10122891, 1:40
MSH6, EPR3945, Abcam, ab92471, GR262215-21, 1:400
β2m, EP2978Y, Abcam, Ab75853, GR3275781-2, 1:100
HLA class I, HCA2, Nordic-MUbio, MUB2036P, 8152b, 1:3200
HLA class I, HC10, Nordic-MUbio, MUB2037P, 818b, 1:3200

Immunohistochemistry tumor tissue samples NICHE study:
Antibody, Clone, Supplier, Catalog number, Lot number, Dilution
β2m, D8P1H, Cell Signaling, 12851, 4, 1:1500
HLA class I, HCA2, Nordic-MUbio, MUB2036P, 7302, 1:5000
HLA class I, HC10, Nordic-MUbio, MUB2037P, 8108B, 1:20000

Imaging mass cytometry:
Antibody, Metal, Clone, Supplier, Catalog number, Lot number, Incubation time (temperature), Dilution
β-catenin, 89Y, D10A8, CST, 8480BF, 8, Overnight (4°C), 1:100
CD103, 168 Er, EPR4166(2), Abcam, ab221210, GR3355784-7, 5h (RT), 1:50
CD11b, 144 Nd, D6X1N, CST, 49420BF, 4, 5h (RT), 1:100
CD11c, 176 Yb, EP1347Y, Abcam, ab216655, GR3357092-9, 5h (RT), 1:100
CD14, 163 Dy, D7A2T, CST, 56082BF, 2, 5h (RT), 1:100
CD15, 171 Yb, MC480, CST, 4744BF, 5, Overnight (4°C), 1:100
CD163, 173 Yb, EPR14643-36, Abcam, 93498BF, Not available, 5h (RT), 1:50
CD20, 142 Nd, E7B7T, CST, 48750BF, 9179056, Overnight (4°C), 1:100
CD204, 164 Dy, J5HTR3, Thermo Fisher Scientific, 14-9054-95, 4338161, 5h (RT), 1:50
CD3, 153 Eu, EP449E, Abcam, ab271850, GR3341846-3, Overnight (4°C), 1:50
CD31, 147 Sm, 89C2, CST, 3528BF, Not available, Overnight (4°C), 1:100
CD38, 169 Tm, EPR4106, Abcam, ab226034, GR3378690-1, Overnight (4°C), 1:100
CD39, 157 Gd, EPR20627, Abcam, ab236038, GR3274485-6, 5h (RT), 1:100
CD4, 145 Nd, EPR6855, Abcam, ab181724, GR3285644-10, Overnight (RT), 1:100
CD45 , 149 Sm, D9M8I, CST, 13917BF, 11, Overnight (4°C), 1:50
CD45RO, 165 Ho, UCHL1, CST, 55618BF, 2, Overnight (4°C), 1:100
CD56, 167 Er, E7X9M, CST, 99746BF, 2, 5h (RT), 1:100
CD57, 151 Eu, HNK-1 / Leu-7, Abcam, ab269781, GR3373313-3, Overnight (4°C), 1:100
CD68, 143 Nd, D4B9C, CST, 76437BF, 2, Overnight (4°C), 1:100
CD7, 174 Yb, EPR4242, Abcam, ab230834, Not available, 5h (RT), 1:100
CD8α, 146 Nd, D8A8Y, CST, 85336BF, Not available, 5h (RT), 1:50
Cleaved caspase-3, 172 Yb, 5A1E, CST, 9664BF, 24, 5h (RT), 1:100
D2-40, 166 Er, D2-40, BioLegend, 916606, B316467, Overnight (4°C), 1:100
FOXP3, 159 Tb, D608R, CST, 12653BF, 8, Overnight (4°C), 1:50
Granzyme B, 150 Nd, D6E9W, CST, 46890BF, 3, 5h (RT), 1:100

Histone H3, 209, D1H2, CST, 4499BF, Not available, Overnight (4°C), 1:50
HLA-DR, 141 Pr, TAL 1B5, Abcam, ab176408, GR3384096-1, 5h (RT), 1:100
ICOS, 161 Dy, D1K2TTM, CST, 89601BF, 4, 5h (RT), 1:50
IDO, 162 Dy, D5J4ETM, CST, 86630BF, 7, Overnight (4°C), 1:100
Ki-67, 152 Sm, 8D5, CST, 9449BF, 11, Overnight (4°C), 1:100
LAG-3, 155 Gd, D2G40TM, CST, 15372BF, Not available, 5h (RT), 1:50
p16ink4a, 175 Lu, D3W8G, CST, 92803BF, 2, Overnight (4°C), 1:100
Pan-keratin, 198 Pt, C11 and AE1/AE3, CST / BioLegend, 4545BF / 914204, 12/B302316, Overnight (4°C), 1:50
PD-1, 160 Gd, D4W2J, CST, 86163BF, 7, 5h (RT), 1:50
PD-L1, 156 Gd, E1L3NR, CST, 13684BF, 17, Overnight (4°C), 1:50
T-bet, 170 Er, 4B10, BioLegend, 644825, B298378, 5h (RT), 1:50
TCRδ, 148 Nd, H41, Santa Cruz, sc-100289, D3021, Overnight (RT), 1:50
TGFβ, 115 In, TB21, Thermo Fisher Scientific, MA5-16949, 151471, 5h (RT), 1:100
TIM-3, 154 Sm, D5D5RTM, CST, 45208BF, 9, 5h (RT), 1:100
Vimentin, 194 Pt, D21H3, CST, 5741BF, 9, Overnight (4°C), 1:50
VISTA, 158 Gd, D1L2GTM, CST, 64953BF, 7, 5h (RT), 1:100
Donkey anti-rabbit IgG, ab6701, Abcam, GR3215731-15, 1 ug/mL
Goat anti-mouse IgG, ab6708, Abcam, GR3300461-15, 1 ug/mL

FACS sorting for scRNA-sequencing:
Antibody, Fluorochrome, Clone, Supplier, Catalog number, Lot number, Dilution
CD3, PE, SK7, BD Biosciences, 345765, 9238465, 1:50
CD45, PerCP-Cy5.5, 2D1, eBioscience, 45-9459-42, 4334219, 1:160
CD7, APC, 124-1D1, eBioscience, 17-0079-42, E12980-101, 1:200
EPCAM, FITC, HEA-125, Miltenyi, 130-098-113, 5161121293, 1:60
TCRγδ, BV421, 11F2, BD Biosciences, 744870, 9340519, 1:80
Live/dead, nIR, n.a., Life Technologies, L10119, 1808830, 1:1000

CITE-sequencing and cell hashing for scRNA-sequencing:
Antibody, Barcode, Clone, Supplier, Catalog number, Lot number, Concentration
TotalSeq-C CD298/β2M (1), GTCAACTCTTTAGCG, LNH-94/2M2, BioLegend, 394661, B264730, 0.5 μg
TotalSeq-C CD298/β2M (6), GGTTGCCAGATGTCA, LNH-94/2M2, BioLegend, 394671, B264724, 0.5 μg
TotalSeq-C CD298/β2M (7), TGTCTTTCCTGCCAG, LNH-94/2M2, BioLegend, 394673, B264730, 0.5 μg
TotalSeq-C CD298/β2M (8), CTCCTCTGCAATTAC, LNH-94/2M2, BioLegend, 394675, B264722, 0.5 μg
TotalSeq-C CD298/β2M (9), CAGTAGTCACGGTCA, LNH-94/2M2, BioLegend, 394677, B264720, 0.5 μg
TotalSeq-C CD45RA, TCAATCCTTCCGCTT, HI100, BioLegend, 304163, B301193, 1 μg
TotalSeq-C CD45RO, CTCCGAATCATGTTG, UCHL1, BioLegend, 304259, B294712, 1 μg

FACS sorting for cell culturing:
Antibody, Fluorochrome, Clone, Supplier, Catalog number, Lot number, Dilution
CD103, FITC, Ber-ACT8, BD Biosciences, 550259, 2332847, 1:10
CD3, Am Cyan, SK7, BD Biosciences, 339186, 9073920, 9161745, 1:20
CD38, PE-Cy7, HIT2, eBioscience, 25-0389-42, 4319912, 1:200
CD39, APC, A1, BioLegend, 328210, B249211, 1:60
CD45RA, PE-Dazzle594, HI100, Sony, 2120730, 126470, 1:20
CD45RO, PerCP-Cy5.5, UCHL1, Sony, 2121110, 138351, 1:20
PD-1, PE, MIH4, eBioscience, 12-9969-42, 1952441, 1:30
TCR α/β, PE-Cy7, IP26, BioLegend, 306720, B303059, 1:40
TCRγδ, BV421, 11F2, BD Biosciences, 744870, 9340519, 1:80
TCR Vδ1, FITC, TS8.2, Invitrogen, TCR2730, UH286015, 1:50
TCR Vδ2, PerCP-Cy5.5, B6, BioLegend, 331424, B279957, 1:200
Live/dead, nIR, n.a., Life Technologies, L10119, 1808830, 1:1000

Immunophenotyping by flow cytometry:
Antibody, Fluorochrome, Clone, Supplier, Catalog number, Lot number, Dilution
CD16, PE, B73.1, BD Biosciences, 332779, 9045985, 1:60
CD103, FITC, Ber-ACT8, BD Biosciences, 550259, 2332847, 1:10
CD122/IL-2Rβ, BV421, TU27, BioLegend, 339010, B313155, 1:20
CD132/IL-2Rγ, APC, TUGh4, BioLegend, 338608, B293032, 1:80
CD161, BV605, DX12, BD Biosciences, 563863, 7030586, 1:20
CD25/IL-2Rα, PE-Cy7, M-A251, BD Biosciences, 557741, 9301660, 1:25
CD215/IL-15Rα, PE, JM7A4, BioLegend, 330208, B265801, 1:80
CD226/DNAM-1, BV510, DX11, BD Biosciences, 742494, 9203072, 1:150
CD3, Am Cyan, SK7, BD Biosciences, 339186, 9161745, 1:20
CD32, APC, FLI8.26, BD Biosciences, 559769, 184743, 1:20
CD38, PE-Cy7, HIT2, eBioscience, 25-0389-42, 4319912, 1:200
CD39, APC, A1, BioLegend, 328210, B249211, 1:60
CD45RA, FITC, L48, BD Biosciences, 335039, 8227525, 1:30
CD45RA, PE-Dazzle594, HI100, Sony, 2120730, 126470, 1:20
CD45RO, PerCP-Cy5.5, UCHL1, Sony, 2121110, 138351, 1:20
CD56, APC-R700, NCAM16.2, BD Biosciences, 565139, 5251693, 1:150
CD64, FITC, 10.1, BD Biosciences, 555527, 58058, 1:20
CD69, PerCP-Cy5.5, FN50, BioLegend, 310925, B266970, 1:200
CD8α, BV605, SK1, BD Biosciences, 564115, 7092, 1:100
CD94, BV605, HP-3D9, BD Biosciences, 743950, 7138571, 1:200
GITR, PE, 108-17, BioLegend, 371204, B244963, 1:50

Granzyme B, PE, GB11, eBiosciences, 12-8899-41, 1928380, 1:50
KIR2DL1, PE, HP3-E4, BD Biosciences, 556063, 86798, 1:20
KIR2DL1/S1, PE, EB6, Beckman Coulter, A09778, 12, 1:50
KIR2DL2/L3/S2, PE, GL183, Beckman Coulter, IM2278U, 200051, 1:50
KIR2DL4, PE, 181703, R&D Systems, FAB2238P, AAHO0209081, 1:10
KIR2DS4, PE, FES172, Beckman Coulter, IM3337, 200037, 1:40
KIR3DL1, PE, DX9, BD Biosciences, 555967, 121769, 1:80
KIR3DL1/S1, PE, Z27, Beckman Coulter, IM3292, 200044, 1:20
KIR3DL2, PE, #539304, R&D Systems, FAB2878P, ADBO0217051, 1:10
LAG3, PE-Cy7, 11C3C65, BioLegend, 369309, B289009, 1:100
NKG2A, APC, z199, Beckman Coulter, A60797, 200046, 1:30
NKG2C, PE, 134591, Beckman Coulter, FAB138P, LCN0818011, 1:20
NKG2D, PE-Cy7, 1D11, BD Biosciences, 562365, 9045733, 1:300
NKp44, APC, P44-8, BioLegend, 325109, B160899, 1:20
NKp46, PE, 9E2, BioLegend, 331907, B150121, 1:20
PD-1, PE, MIH4, eBioscience, 12-9969-42, 1952441, 1:30
Perforin, PE-Cy7, dG9, BioLegend, 308125, B215704, 1:20
TCRγδ, BV421, 11F2, BD Biosciences, 744870, 9340519, 1:80
TCRγδ, BV650, 11F2, BD Biosciences, 745359, 7222894, 1:40
TCR Vδ1, FITC, TS8.2, Invitrogen, TCR2730, UH286015, 1:50
TCR Vδ2, PerCP-Cy5.5, B6, BioLegend, 331424, B279957, 1:200
TIGIT, APC, 1D11, BD Biosciences, 562365, 9045733, 1:300
Live/dead, nIR, n.a., Life Technologies, L10119, 1808830, 1:1000

Characterization of cancer cell lines:
Antibody, Fluorochrome, Clone, Supplier, Catalog number, Lot number, Dilution
β2m, PE, 2M2, BioLegend, 316305, B326396, 1:100
CD112, PE, R2.525, BD Biosciences, 551057, 11886, 1:10
CD155, PE, 300907, R&D Systems, FAB25301P, AANQ0108091, 1:10
CD277/BTN3A1, PE, BT3.1, Miltenyi, 130-117-693, 5201109412, 1:50
HLA-A/B/C, FITC, W6/32, eBioscience, 11-9983-41, 4291421, 1:100
HLA-A/B/C, AF647, W6/32, BioLegend, 311414, 2132555, 1:160
HLA-E, BV421, 3D12, BioLegend, 342611, B296865, 1:20
HLA-G, APC, 87G, BioLegend, 335909, B297169, 1:20
MICA/B, PE, 6D4, BioLegend, 320906, B279674, 1:300
ULBP1, PE, 170818, R&D Systems, FAB1380P, AAJW0419061, 1:10
ULBP2/5/6, PE, 165903, R&D Systems, FAB1298P, LWE0718071, 1:20
ULBP3, PE, 166510, R&D Systems, FAB1517P, ABPX0719061, 1:20
ULBP4, PE, 709116, R&D Systems, FAB6285P, ADXP0420021, 1:20
Isotype control mouse IgG1, PE, MOPC-21, BD Biosciences, 556650, 65215
Isotype control mouse IgG2a, PE, G155-178, BD Biosciences, 556653, 8121794
Isotype control mouse IgG2a, FITC, BD Biosciences, 349051, 6194980
Isotype control mouse IgG2b, PE, 133303, R&D Systems, IC0041P, LHG1919091
Live/dead, nIR, n.a., Life Technologies, L10119, 1808830, 1:1000

Characterization of organoids:
Antibody, Fluorochrome, Clone, Supplier, Catalog number, Lot number, Dilution
β2m, FITC, 2M2, BioLegend, 316304, B261327, 1:100
HLA-A,B,C, PE, W6/32, ThermoFisher, MA1-19027, 7096730, 1:20
PD-L1, APC, MIH1, eBioscience, 17-5983-42, 4307992, 1:200
Isotype mouse IgGκ, APC, P36281, eBioscience, 17471442, 1943772, 1:200
Isotype mouse IgGκ, FITC, MOPC-21, BD Bioscience, 555748, 1054855, 1:100
Isotype mouse IgGκ, PE, MOPC-31C, BD Bioscience, 550617, 0065215, 1:20
Live/dead, nIR, n.a., Life Technologies, L10119, 2184311, 1:2000

Reactivity assay with cancer cell lines:
Antibody, Fluorochrome, Clone, Supplier, Catalog number, Lot number, Dilution
CD137, APC, 4B4-1, BD Biosciences, 550890, 0188145, 1:100
CD226/DNAM-1, BV510, DX11, BD Biosciences, 742494, 9203072, 1:150
CD3, AF700, UCHT1, BD Biosciences, 557943, 3263589, 1:400
CD39, BV421, A1, BioLegend, 328214, B308983, 1:80
CD40L, PE, TRAP1, BD Biosciences, 555700, 0267576, 1:10
NKG2D, PE-Cy7, 1D11, BD Biosciences, 562365, 9045733, 1:300
OX40, FITC, ACT35, BioLegend, 350006, B314952, 1:20
PD-1, PE, MIH4, eBioscience, 12-9969-42, 1952441, 1:30
TCRγδ, BV650, 11F2, BD Biosciences, 745359, 7222894, 1:40
Live/dead, nIR, n.a., Life Technologies, L10119, 1808830, 1:1000

Reactivity assay with organoids:
Antibody, Fluorochrome, Clone, Supplier, Catalog number, Lot number, Dilution
CD107a, FITC, H4A3, BioLegend, 328605, B27833, 1:50
CD28, - , CD28.2, eBioscience, 16028981, 2310226, 1:200 (coating)
CD3, PerCP-Cy5.5, SK7, eBioscience, 332771, 0203984, 1:20
CD4, FITC, RPA-T4, BD Bioscience, 555346, 9073869, 1:20
CD8, BV421, RPA-T8, BD Bioscience, 562429, 7082750, 1:200
IFNγ, APC, B27, BD Bioscience, 554702, 0332147, 1:40

TCRγδ, PE, 11F2, BD Bioscience , 333141, 0070146, 1:20
Live/dead, nIR, n.a., Life Technologies, L10119, 2184311, 1:2000

Blocking antibodies:
Antibody, Fluorochrome, Clone, Supplier, Catalog number, Lot number, Working concentration
DNAM-1, Purified, DX11, BD Biosciences, 559786, 0016942, 3 μg/mL
MICA/MICB, Purified, 6D4, BioLegend, 320919, B321272, 12 μg/mL
TCRγδ, Purified, 5A6.E9, Invitrogen, TCR1061, VA288448, 3 μg/mL
ULBP1, Purified, 170818, R&D Systems, MAB1380-100, JHI0317071, 1 μg/mL
ULBP2/5/6, Purified, 165903, R&D Systems, MAB1298-100, JQE0420031, 3 μg/mL
ULBP3, Purified, 166510, R&D Systems, MAB1517-100, JFY0219122, 6 μg/mL

Validation

All antibodies were validated and titrated to determine their optimal concentration, as explained in more detail below.

The mismatch repair (MMR), HLA class I, and β2m status were determined by immunohistochemical (IHC) staining. MMR-deficiency was defined as the lack of expression of at least one of the MMR-proteins by tumor cells in the presence of an internal positive control. Immunohistochemical detection of HLA class I expression on tumors was performed with the monoclonal HLA class I antibodies HCA2 and HC10, and classified as HLA class I positive when membranous staining of both HCA2 and HC10 was observed , weak, when membranous staining was observed but in lower levels compared to the immune-infiltration, or loss, when one or both HCA2 and HC10 do not show membranous staining, as validated previously (Ijsselsteijn et al, Revisiting immune escape in colorectal cancer in the era of immunotherapy, Br J Cancer, 2019). β2m expression was defined as positive, negative or aberrant expression in tumor cells in the presence of a positive internal control. All tissue sections were scored by a pathologist.

For imaging mass cytometry, the specificities of unconjugated antibodies was first tested by immunohistochemical (IHC) staining. Thereafter, the antibodies were conjugated to their respective metal and validated once more by IHC staining, of which the results were compared to the pre-conjugation IHC results. Antibodies were then tested by imaging mass cytometry on FFPE tonsil sections or colorectal cancer sections as positive control. The optimal staining protocol and antibody specificities have been previously validated and described by Ijsselsteijn et al. A 40-Marker Panel for High Dimensional Characterization of Cancer Immune Microenvironments by Imaging Mass Cytometry, Frontiers in Immunology, 2019.

The specificities of flow cytometry antibodies were validated by the suppliers through flow cytometry staining versus isotype controls. Detailed information and references of the antibodies can be found on https://www.bdbiosciences.com, https://www.biolegend.com, https://www.thermofisher.com, https://www.beckmancoulter.com, https://www.rndsystems.com, https://www.sonybiotechnology.com, or https://www.miltenyibiotec.com by searching using the catalog number of the antibodies listed above on the supplier's website. We further validated the antibody staining patterns on PBMC control samples with or without stimulation with PMA/ionomycin. In the flow cytometry experiments, isotype and/or FMO control samples were included.

Antibodies used for the characterization and reactivity assay with organoids:
The following antibodies have been previously used and described in Dijkstra et al. Cell 2018 and Cattaneo et al. Nature Protocols 2020 (Dijkstra et al. Generation of Tumor-Reactive T Cells by Co-culture of Peripheral Blood Lymphocytes and Tumor Organoids, Cell, Volume 174, Issue 6, 2018, doi.org/10.1016/j.cell.2018.07.009; Cattaneo et al. Tumor organoid–T-cell coculture systems, Nat Protoc 15, 15–39 (2020), doi.org/10.1038/s41596-019-0232-9):
Antibody, Fluorochrome, Clone, Supplier, Catalog number, Lot number, Dilution
HLA-A,B,C, PE, W6/32, ThermoFisher, MA1-19027, 7096730, 1:20
PD-L1, APC, MIH1, eBioscience, 17-5983-42, 4307992, 1:200
Isotype mouse IgGκ, APC, P36281, eBioscience, 17471442, 1943772, 1:200
Isotype mouse IgGκ, FITC, MOPC-21, BD Bioscience, 555748, 1054855, 1:100
Isotype mouse IgGκ, PE, MOPC-31C, BD Bioscience, 550617, 0065215, 1:20
CD107a, FITC, H4A3, BioLegend, 328605, B27833, 1:50
CD28, -, CD28.2, eBioscience, 16028981, 2310226, 1:200 (coating)
CD3, PerCP-Cy5.5, SK7, eBioscience, 332771, 0203984, 1:20
CD4, FITC, RPA-T4, BD Bioscience, 555346, 9073869, 1:20
CD8, BV421, RPA-T8, BD Bioscience, 562429, 7082750, 1:200
IFNγ, APC, B27, BD Bioscience, 554702, 0332147, 1:40
Live/dead, nIR, n.a., Life Technologies, L10119, 2184311, 1:2000

The following antibody has been referenced by Zhu et al. Nat Commun. 2019 (Zhu et al. Precisely controlling endogenous protein dosage in hPSCs and derivatives to model FOXG1 syndrome. Nat Commun. 2019 Feb 25;10(1):928. doi: 10.1038/s41467-019-08841-7):
β2m, FITC, 2M2, BioLegend, 316304, B261327, 1:100

Eukaryotic cell lines

Policy information about cell lines

Cell line source(s)

All cancer cell lines were derived from human: HCT-15 from colon adenocarcinoma, LoVo from colon adenocarcinoma (derived from metastatic site: left supraclavicular lymph node), HT-29 from colon adenocarcinoma, SW403 from colon adenocarcinoma, SK-CO-1 from colon adenocarcinoma (derived from metastatic side: ascites), Daudi from Burkitt lymphoma, and K-562 from chronic myeloid leukemia. All cancer cell lines derived from the ATCC.

Authentication

All cancer cell lines were authenticated by STR profiling.

Mycoplasma contamination

All cancer cell lines were tested for mycoplasma. None of the cell lines used in this study tested positive for mycoplasma.

Commonly misidentified lines
(See ICLAC register)

No commonly misidentified lines were used in this study.

Human research participants

Policy information about studies involving human research participants

Population characteristics

DRUP study cohort:
The DRUP is a national, prospective, non-randomized, multi-drug and multi-tumor study, designed and conducted on behalf of the Center for Personalized Cancer Treatment (CPCT). Patients who were eligible for the study had an advanced or metastatic solid tumor, multiple myeloma or B cell non-Hodgkin lymphoma, and had exhausted standard-treatment options. A tumor genetic or protein-expression test (CPCT or regular diagnostics) had to have revealed a potentially actionable variant, for which FDA- and/or EMA-approved targeted therapy was available—but not for the tumor type in question. Across all completed and ongoing DRUP study cohorts, we performed a preliminary analysis on all patients with MMR-d tumors treated with PD-1 blockade with available WGS, RNA-seq and clinical outcome data. Detection of MMR-d by standard of care diagnostics and conformation by WGS was required for inclusion in this analysis. At inclusion in the DRUP, patients were required to be ≥18 years of age, with acceptable organ function and performance status (Eastern Cooperative Oncology Group (ECOG) score ≤ 2), and to have objectively evaluable disease of which a fresh baseline tumor biopsy could safely be obtained. Detailed baseline characteristics of this cohort are provided in Supplemental Table 1. In total, 32 out of 71 (45%) patients were female. The median (IQR) age was 68 (57-74) years.

Hartwig cohort:
We analyzed 2,256 metastatic tumors included in the freely available Hartwig database (Priestley et al, 2019), which (i) were MMR-p (WGS-based MSIseq6 score ≤4), (ii) had available WGS data passing standard quality controls (as defined before by Priestley et al, 2019, including a sequencing-based tumor purity of at least 20%), (iii) and had available RNA-seq data. Extensive details on the consortium and the full patient cohort has been published previously (Priestley et al, 2019). In brief, the CPCT-02 consortium was established to collect tumor biopsies of patients with advanced stage solid malignancies, in order to analyze the cancer genome by WGS and to discover predictors for systemic treatment outcome. Patients eligible for inclusion were at least 18 years and had locally advanced or metastasized solid tumors. Condition for enrollment was the possibility to safely obtain a histological biopsy from a metastasis or primary tumor prior to the start of a new line of systemic treatment. Patient-level clinical characteristics of this cohort can be found in Supplemental Table 3.

Leiden IMC cohort:
Primary colon cancer tissues were from 17 patients with colon cancer who underwent surgical resection of their tumor at the Leiden University Medical Center (LUMC, the Netherlands). Clinical characteristics of these patients are outlined in Supplemental Table 4. In total, 10 out of 17 (59%) patients were female. The median (range) age was 68 (36-82) years.

NICHE study cohort:
In addition, primary colon cancer tissues from 10 patients with colon cancer included in the NICHE study (NCT03026140) carried out at the Netherlands Cancer Institute (NKI, the Netherlands) were used for this study. A detailed description of the NICHE samples has recently been published by us in a sister journal, Nature Medicine (Chalabi, M. et al. Neoadjuvant immunotherapy leads to pathological responses in MMR-proficient and MMR-deficient early-stage colon cancers. Nat Med 26, 566-576, doi:10.1038/s41591-020-0805-8 (2020)). We have referred to this study in this manuscript.

ITO study cohort:
In the ITO (Immunogenicity of Tumor Organoids) study, tumor organoids were derived from two patients with MMR-d colorectal cancer included in the study at the Netherlands Cancer Institute (NKI, The Netherlands). Mismatch repair deficiency was confirmed by immunohistochemical staining for the mismatch repair proteins MSH2, MSH6, MLH1 and PMS2 in routine assessment by a pathologist. Tumor organoids generated from this study have been previously described by Dijkstra et al. (Dijkstra et al. Generation of Tumor-Reactive T Cells by Co-culture of Peripheral Blood Lymphocytes and Tumor Organoids, Cell, Volume 174, Issue 6, 2018, doi.org/10.1016/j.cell.2018.07.009). We have referred to this study in this manuscript.

Patient-derived tumor organoids:
PDTO-1 was derived from resection material of a primary colorectal cancer tumor. PDTO-2 was derived from a biopsy of a peritoneal metastasis of the colorectal cancer. Procedures performed with patient specimens were approved by the Medical Ethical Committee of the Netherlands Cancer Institute – Antoni van Leeuwenhoek hospital (study NL48824.031.14) and written informed consent was obtained from all patients. All PDTOs were authenticated by SNP or STR profiling. All PDTOs were tested for mycoplasma. None of the organoids used in this study tested positive for mycoplasma.

Recruitment

DRUP study cohort:
Patients with MMR-d cancers of various anatomical origins who exhausted all regular treatment options were recruited in 22 Dutch hospitals participating in the DRUP trial. Patients who were eligible for the study had an advanced or metastatic solid tumor, multiple myeloma or B cell non-Hodgkin lymphoma, and had exhausted standard-treatment options. A tumor genetic or protein-expression test (CPCT or regular diagnostics) had to have revealed a potentially "actionable variant", for which FDA- and/or EMA-approved targeted therapy was available—but not for the tumor type in question. In case of the subgroup of DRUP-included patients analyzed in our study, the "actionable variant" was microsatellite instability. In addition, patients were required to be ≥18 years of age, with acceptable organ function and performance status (Eastern Cooperative Oncology Group (ECOG) score ≤ 2), and to have objectively evaluable disease of which a fresh baseline tumor biopsy could safely be obtained. A detailed description of patient recruitment in the DRUP study can be found in the previous publication by us in this journal (van der Velden, D. L. et al. The Drug Rediscovery protocol facilitates the expanded use of existing anticancer drugs. Nature 574, 127-131, doi:10.1038/s41586-019-1600-x (2019).

Hartwig cohort:
Patients were included in 42 academic, teaching, and general hospitals across the Netherlands, under the protocol of the

	Center for Personalized Cancer Treatment (CPCT) consortium (CPCT-02 Biopsy Protocol, ClinicalTrial.gov no. NCT01855477). A detailed description of patient recruitment in the Hartwig dataset can be found in the previous publication by us in this journal (Priestley, D. L. et al. Pan-cancer whole-genome analyses of metastatic solid tumours. Nature 575, pages 210–216, doi:10.1038/s41586-019-1689-y (2019)).<br><br>Leiden IMC cohort:<br>The LUMC IMC cohort constitutes a consecutive cohort of treatment-naive patients with colon cancer who underwent surgical resection of their tumor at the Leiden University Medical Center (LUMC, the Netherlands).<br><br>NICHE study cohort:<br>Recruitment of patients included in the NICHE study can be found in the previous publication by us in a sister journal, Nature Medicine (Chalabi, M. et al. Neoadjuvant immunotherapy leads to pathological responses in MMR-proficient and MMR-deficient early-stage colon cancers. Nat Med 26, 566-576, doi:10.1038/s41591-020-0805-8 (2020)). We have referred to this study in this manuscript.<br><br>ITO study cohort:<br>Patients were accrued at the Netherlands Cancer Institute. Eligible patients were those with colorectal, gastric or non-small cell lung cancer that were pre-planned to undergo a study-related or standard of care biopsy procedure/tumor resection, during which an extra tissue specimen was obtained for tumor organoid generation.
Ethics oversight	The DRUP study and the generation of the Hartwig database were initiated and conducted on behalf of the Center for Personalized Cancer Treatment (CPCT; clinicaltrials.gov: NCT02925234, NCT01855477). These studies were approved by the Medical Ethical Committee of the Netherlands Cancer Institute in Amsterdam and the University Medical Center Utrecht, respectively, and were conducted in accordance with good clinical practice guidelines and the Declaration of Helsinki's ethical principles for medical research. Written informed consent was obtained from all study subjects. This study was approved by the Medical Ethical Committee of the Leiden University Medical Center (protocol P15.282), and patients provided written informed consent. Details on the protocol of the NICHE study have been described in the previous publication by us in a sister journal, Nature Medicine (Chalabi, M. et al. Neoadjuvant immunotherapy leads to pathological responses in MMR-proficient and MMR-deficient early-stage colon cancers. Nat Med 26, 566-576, doi:10.1038/s41591-020-0805-8 (2020)). We have referred to this study in this manuscript. The ITO study (Dutch Central Committee on Research Involving Human Subjects number: NL48824.031.14) was approved by the Medical Ethical Committee of the Netherlands Cancer Institute – Antoni van Leeuwenhoek hospital and written informed consent was obtained from all patients.

Note that full information on the approval of the study protocol must also be provided in the manuscript.

Clinical data

Policy information about clinical studies

All manuscripts should comply with the ICMJE guidelines for publication of clinical research and a completed CONSORT checklist must be included with all submissions.

Clinical trial registration	DRUP: NCT02925234; CPCT study: NCT01855477; NICHE trial: NCT03026140
Study protocol	The study protocols will be made available upon request.
Data collection	Patients in the DRUP study were accrued at 22 hospitals throughout the Netherlands between 2016 and 2021. Recruitment and data collection involved dozens of medical specialists and trained research nurses, both at the site of inclusion as at the sites of coordination (Netherlands Cancer Institute, Amsterdam Medical Center, Leiden University Medical Center), which minimizes self-selection biases. Radiological response evaluations were performed according to RECIST 1.1 criteria at the site of accrual. A detailed description of the data collection of the DRUP study can be found in the previous publication by us in this journal (van der Velden, D. L. et al. The Drug Rediscovery protocol facilitates the expanded use of existing anticancer drugs. Nature 574, 127-131, doi:10.1038/s41586-019-1600-x (2019).<br><br>Patients in the Hartwig dataset were included in 42 academic, teaching, and general hospitals across the Netherlands between February 2016 and December 2019. A detailed description of data collection in the Hartwig dataset can be found in the previous publication by us in this journal (Priestley, D. L. et al. Pan-cancer whole-genome analyses of metastatic solid tumours. Nature 575, pages 210–216, doi:10.1038/s41586-019-1689-y (2019).<br><br>A detailed description of the data collection of the NICHE study can be found in the previous publication by us in a sister journal, Nature Medicine (Chalabi, M. et al. Neoadjuvant immunotherapy leads to pathological responses in MMR-proficient and MMR-deficient early-stage colon cancers. Nat Med 26, 566-576, doi:10.1038/s41591-020-0805-8 (2020)). We have referred to this study in this manuscript.
Outcomes	For DRUP-based analyses, we used clinical benefit (defined as at least 4 months of disease control) as the primary outcome, as predefined in the study protocol. Additionally, we reported the best overall response, which was defined as a secondary outcome measure in the study protocol. Following RECIST1.1 criteria, these outcomes were assessed by the local treatment team at the site of accrual. Consistent with the study protocol, we considered these outcomes evaluable in patients who received at least two cycles of intravenous study medication, and for whom response was radiologically or clinically evaluable (at the treating physician's discretion). A detailed description of the outcomes of the DRUP study can be found in the previous publication by us in this journal (van der Velden, D. L. et al. The Drug Rediscovery protocol facilitates the expanded use of existing anticancer drugs. Nature 574, 127-131, doi:10.1038/s41586-019-1600-x (2019).<br><br>A detailed description of primary objectives, safety and feasibility, and secondary and translational endpoints of the NICHE study can be found in the previous publication by us in a sister journal, Nature Medicine (Chalabi, M. et al. Neoadjuvant immunotherapy leads to pathological responses in MMR-proficient and MMR-deficient early-stage colon cancers. Nat Med 26, 566-576, doi:10.1038/

s41591-020-0805-8 (2020)). We have referred to this study in this manuscript.

Flow Cytometry

Plots

Confirm that:

☒ The axis labels state the marker and fluorochrome used (e.g. CD4-FITC).

☒ The axis scales are clearly visible. Include numbers along axes only for bottom left plot of group (a 'group' is an analysis of identical markers).

☒ All plots are contour plots with outliers or pseudocolor plots.

☒ A numerical value for number of cells or percentage (with statistics) is provided.

Methodology

Sample preparation	γδ T cells were isolated from MMR-deficient colon cancer tissues and expanded in vitro for functional assays. Reactivity of γδ T cells was tested by co-culturing with cancer cell lines and organoids, after which the γδ T cells were collected for analysis.
Instrument	For experiments at the LUMC, flow cytometry data were acquired with a FACS Canto II 3L (BD Biosciences), FACS LSR Fortessa 4L (BD Biosciences), and FACS LSR Fortessa X-20 4L (BD Biosciences). For experiments at the Netherlands Cancer Institute, flow cytometry data were acquired with a BD LSRFortessa™ Cell Analyzer SORP (BD Biosciences).
Software	For experiments at the LUMC, FACSDiva software version 9.0 (BD Biosciences) and FlowJo software version 10.6.1 (Tree Star Inc). For experiments at the Netherlands Cancer Institute, FACSDiVa 8.0.2 (BD Biosciences) was used.
Cell population abundance	γδ T cells accounted for 0.5-8.1% of the single, live lymphocyte population in the tumor samples at the time of FACS-sorting. The purity of γδ T cells was evaluated after culturing by flow cytometry and was >98%.

Gating strategy

In Figure 3a and Extended Data Fig. 5a:
1) FSC-A versus SSC-A gated on lymphocytes
2) FSC-A versus FSC-H gated on single cells
3) Live/dead-nIR versus SSC-A gated on live cells
4a) CD3-Am Cyan versus TCRγδ-BV421 gated on TCRγδ+ CD3+ cells
4b) CD3-Am Cyan versus TCRγδ-BV650 gated on TCRγδ+ CD3+ cells for detection of cytokine receptors (CD122, CD25, CD215, CD132)
5) Indicated markers for immunophenotyping versus TCRγδ-BV421 (or TCRγδ-BV650) for detection of marker-positive cells (see Supplemental Table 7)

In Figure 3b and Extended Data Fig. 5b:
1) FSC-A versus SSC-A gated on lymphocytes
2) FSC-A versus FSC-H gated on single cells
3) Live/dead-nIR versus SSC-A gated on live cells
4) CD112-PE versus SSC-A gated on CD112+ target cells, CD155-PE versus SSC-A gated on CD155+ target cells, CD277-PE versus SSC-A gated on CD277+ target cells, HLA-A/B/C-FITC versus SSC-A gated on HLA-A/B/C+ target cells, HLA-E-BV421 versus SSC-A gated on HLA-E+ target cells, HLA-G-APC versus SSC-A gated on HLA-G+ target cells, MICA/B-PE versus SSC-A gated on MICA/B+ target cells, ULBP1-PE versus SSC-A gated on ULBP1+ target cells, ULBP2/5/6-PE versus SSC-A gated on ULBP2/5/6+ target cells, ULBP3-PE versus SSC-A gated on ULBP3+ target cells, ULBP4-PE versus SSC-A gated on ULBP4+ target cells.

In Figure 3c, Extended Data Fig. 5c,d,f, Extended Data Fig. 8a,b,d:
1) FSC-A versus SSC-A gated on lymphocytes
2) FSC-A versus FSC-H gated on single cells
3) Live/dead-nIR versus SSC-A gated on live cells
4) CD3-AF700 versus TCRγδ-BV650 gated on TCRγδ+ CD3+ cells
5) CD137-APC versus SSC-A gated on CD137+ γδ T cells, OX40-FITC versus SSC-A gated on OX40+ γδ T cells, PD-1-PE versus SSC-A gated on PD-1+ γδ T cells, NKG2D-PE-Cy7 versus SSC-A gated on NKG2D+ γδ T cells

In Figure 3f and 3g:
1) FSC-A versus SSC-A gated on lymphocytes
2) SSC-H versus SSC-A gated on single cells
3) Live/dead-nIR versus CD3-PerCP-Cyanine5.5 gated on CD3+ live cells
4) TCRγδ-PE versus FSC-H gated on TCRγδ+ cells
5) CD4-FITC versus CD8-BV421 gated on CD4- CD8+/- cells
6) IFNγ-APC versus FSC-H gated on IFNγ+ cells

In Figure 3i:
1) FSC-A versus SSC-A gated on lymphocytes
2) SSC-H versus SSC-A gated on single cells
3) Live/dead-nIR versus CD3-PerCP-Cyanine5.5 gated on CD3+ live cells
4) CD8-BV421 versus TCRγδ-PE gated on CD8+/- TCRγδ+ cells
5) IFNγ-APC versus CD107a-FITC gated on IFNγ+ CD107a-, IFNγ+ CD107a+ and IFNγ- CD107+ cells

In Extended Data Fig. 2a:
1) FSC-A versus SSC-A gated on lymphocytes
2) FSC-A versus FSC-W gated on single cells
3) Live/dead-nIR versus SSC-A gated on live cells
4) CD45-PerCp-Cy5.5 versus EpCAM-FITC gated on CD45+ EpCAM– cells
5) CD3-PE versus TCRγδ-BV421 gated on TCRγδ+ CD3+ cells

In Extended Data Fig. 4:
1) FSC-A versus SSC-A gated on lymphocytes
2) FSC-A versus FSC-W gated on single cells
3) Live/dead-nIR versus SSC-A gated on live cells
4) CD3-Am Cyan versus TCRγδ-BV421 gated on TCRγδ+ CD3+ cells
5) PD-1-PE versus TCRγδ-BV421 gated on PD-1– and PD-1+ γδ T cells, where possible

In Extended Data Fig. 6a:
1) FSC-A versus SSC-A gated on cells
2) FSC-A versus FSC-H gated on single cells
3) Live/dead-nIR versus FSC-A gated on live cells
4) B2M-PE as count (histogram)

In Extended Data Fig. 7b:
1) FSC-A versus SSC-A gated on cells
2) SSC-A versus SSC-H gated on single cells
3) Live/dead-nIR versus FSC-H gated on live cells
4) B2M-FITC/MHC-I-PE/PD-L1-APC as count (histogram)

All gating strategies are provided in the Extended Data Figures.

☒ Tick this box to confirm that a figure exemplifying the gating strategy is provided in the Supplementary Information.
